# A Study of Adult Olfactory Proteins of Primitive Ghost Moth, *Endoclita signifer* (Lepidoptera, Hepialidae)

**DOI:** 10.3390/life13122264

**Published:** 2023-11-27

**Authors:** Guipeng Xiao, Jintao Lu, Zhende Yang, Hengfei Fu, Ping Hu

**Affiliations:** 1 Biotechnology, Faculty of Science, Autonomous University of Madrid, 28029 Madrid, Spain; guipeng.xiao@estudiante.uam.es; 2 Guangxi Colleges and Universities Key Laboratory for Cultivation and Utilization of Subtropical Forest Plantation, Guangxi Key Laboratory of Forest Ecology and Conservation, College of Forestry, Guangxi University, Nanning 530004, Chinadzyang68@gxu.edu.cn (Z.Y.)

**Keywords:** antennae, transcriptome, olfactory proteins, *Endoclita signifer*, eucalyptus

## Abstract

*Endoclita signifer* is a prominent wood-boring insect species in eucalyptus plantations in Guangxi, China, causing significant ecological and economic damage. A novel approach to controlling the challenging wood-boring pest involves disrupting the olfactory communication between insects and the volatile compounds emitted by plants. To identify the olfactory proteins contributing to host selection based on 11 GC-EAD-active volatiles from eucalyptus leaves and to discover the highly expressed olfactory proteins, we conducted a study on the antennal transcriptomes of adult *E. signifer* and screened key olfactory proteins in the antennae. We identified a total of 69 olfactory proteins. When compared to the larval transcriptomes, the antennal transcriptome of adult *E. signifer* revealed the presence of 17 new odorant-binding proteins (OBPs), including 2 pheromone-binding proteins (PBPs), 7 previously unreported chemosensory proteins (CSPs), 17 new odorant receptors (ORs), 4 new gustatory receptors (GRs), 11 novel ionotropic receptors (IRs), and 2 sensory neuron membrane proteins (SNMPs). Through the phylogenetic tree of OBPs and ORs, we identified *EsigPBP2* and *EsigPBP3* as two of the three PBPs, designated *EsigOR13* as EsigOrco, and recognized *EsigOR10* and *EsigOR22* as the newly discovered EsigPRs in *E. signifer*. In the adult antennae, the expression levels of *EsigGOBP14*, *EsigGOBP13*, *EsigOBP14*, *EsigOBP17*, *EsigCSP14*, and *EsigOR16* were notably high, indicating that these proteins could be pivotal in binding to plant volatiles.

## 1. Introduction

*Endoclita signifer* Walker (ghost moth, Lepidoptera, Hepialidae) is the major wood-boring pest of eucalyptus that has spread extensively across Guangxi, China, causing substantial damage to eucalyptus trees and ecosystems [1]. As an omnivorous pest, the native host plants of *E. signifer* include 30 families, 40 genera, and 51 species [2]. Interestingly, with the widespread establishment of eucalyptus plantations in Guangxi, *E. signifer* has transitioned from its native host plants to eucalyptus, serving as a notable example of a native pest adapting to introduced hosts. Meanwhile, the third instar larvae of *E. signifer* display a unique behavior pattern: they move from the soil of eucalyptus plantations to standing trees, where they feed on bark and bore into the interior wood. And the phenomenon, along with the damage inflicted by *E. signifer* on eucalyptus trees within one to three years, indicates that both larval [3,4] and adult host selection contribute to its adaptation to eucalyptus. And because the sensitive olfactory system of the insect plays a key role in host selection, mating, and feeding, we hypothesized that the olfactory system of adult *E. signifer* significantly contributes to its host selection, with olfactory proteins serving as the functional components in this process.

The first odorant-binding protein (OBP) was discovered in *Antheraea polyphemus*. It was a small, 15 kD soluble protein uniquely found in the male antennae, capable of binding to labeled pheromones in native gels [5]. OBPs play a pivotal role in the initial stages of odor detection, involving the identification, screening, binding, and transportation of odor molecules. Studies examining their function have revealed their significance in neuronal activation. This was initially demonstrated through research on mutants lacking the OBP LUSH, which exhibited insensitivity to concentrations of the male-specific pheromone 11-cis vaccenyl acetate (cVA), which strongly activates neurons expressing the Or67d receptor subunit in *Drosophila* [6]. Chemosensory proteins (CSPs), unlike OBPs, exhibit a less rigid structure, allowing them to bind ligands with a broader ligand [7], and a study showed that CSPs in coleopteran insects have duplication and differentiation under the role of natural selection [8]. The mechanism of transporting odor molecules from binding proteins to receptors is similar in *Ostrinia furnacalis*. Specifically, the sex pheromones E-12-tetradecenyl acetate and Z-12-tetradecenyl acetate are recognized and bound by the pheromone-binding proteins (PBPs) OfurPBP3 and OfurPBP2. Subsequently, these bound pheromones are transported to the odorant receptors (ORs) OfurOR4 and OfurOR6, leading to their activation [9]. Factually, ORs function as the primary receptors, and they form heteromers with a shared, highly conserved subunit known as the OR coreceptor (Orco) in insects [10,11]. Orco has evolved to act as a coreceptor, enabling a unified mechanism for regulating odorant sensitivity that operates independently of the expression of individual tuning receptors [12]. The first identified pheromone receptor (PR) in *Drosophila* was *Or67d*. It was subsequently discovered that a mutation at amino acid 23 in *Or67d* caused the substitution of cysteine with tryptophan, and mutation Or67d still expressed but was entirely unresponsive to the cVA pheromone [13]. However, putative insect olfactory receptors are not highly conserved among insect species [14]. Indeed, in addition to odorant receptors (ORs), insects also have gustatory receptors (GRs), ionotropic receptors (IRs), and sensory neuron membrane proteins (SNMPs) that function as part of the olfactory receptor system. These various receptor types collectively contribute to the insects’ complex sensory perception, enabling them to detect and respond to a wide range of environmental cues. 

Research in the field of insect olfaction has predominantly centered around behaviors linked to reproduction, including the detection of sex pheromones, mating, and the selection of host and predator volatiles [6,7,8]. In *E. signifer* larvae, our initial investigation focused on the volatiles emitted by eucalyptus trunks and the humus of the forest floor when the third instar larvae transitioned to eucalyptus as their host. We identified a total of 34 volatile compounds, and, among them, alpha-phellandrene stood out as one of the most prominent and volatile herbivore-induced plant volatiles (HIPVs) [4]. Secondly, we screened the function volatile by electrophysiology and behavior choice. We found that 11 volatiles were GC-EAD-active compounds, and o-cymene stimulated significant behavior attraction in third instar *E. signifer* larvae [4]. Finally, through the transcriptome of larvae heads (different instar) and tegument, we identified 62 olfactory proteins [15,16]; furthermore, we demonstrated that EsigGOBP1 was the key protein for binding alpha-phellandrene [17]. All the above results indicated that *E. signifer* larvae possess the ability to select their host, and olfactory proteins play a significant role in this selection process. In adult *E. signifer*, 40 volatile compounds were identified from one- and five-year-old eucalyptus leaves, in which 11 volatiles were GC-EAD-active compounds, and we observed an increasing trend in the electroantennogram (EAG) response as the concentration of the stimulus sample increased (unpublished data). However, we have yet to identify the key olfactory proteins of *E. signifer* adult and elucidate how they contribute to the host selection process.

This study examined the antennal transcriptomes of adult *E. signifer* and analyzed the expression profiles of these transcriptomes, with a particular focus on identifying key proteins associated with the recognition of 11 GC-EAD-active volatiles in adult *E. signifer*. By comparing these key proteins with previous findings, we aimed to uncover the olfactory proteins responsible for recognizing plant volatiles. This research has the potential to offer novel insights into pest control strategies.

## 2. Results

### 2.1. Transcriptome Sequencing and Sequence Assembly

**Figure 1:**
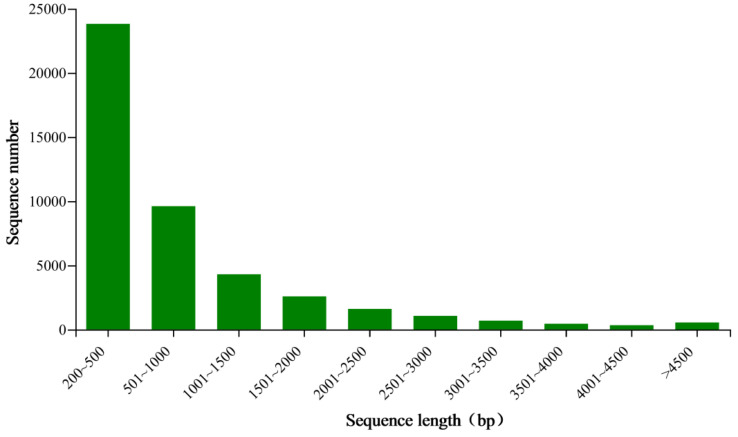
Length distribution of unigenes in the antennal transcriptome of *E. signifer*.

We generated 48 million raw reads from each cDNA library of the *E. signifer* adult, averagely. Approximately 94.09% of these reads exhibited q30 quality scores on average. The number of 44,905 unigenes, with an N50 of 1488 bp, average length of 859 bp, were obtained (Figure 1 and Table 1). Additionally, BUSCO analysis indicated a completion rate of 89.30%.

### 2.2. Homology Analysis and Gene Ontology Annotation

**Figure 2:**
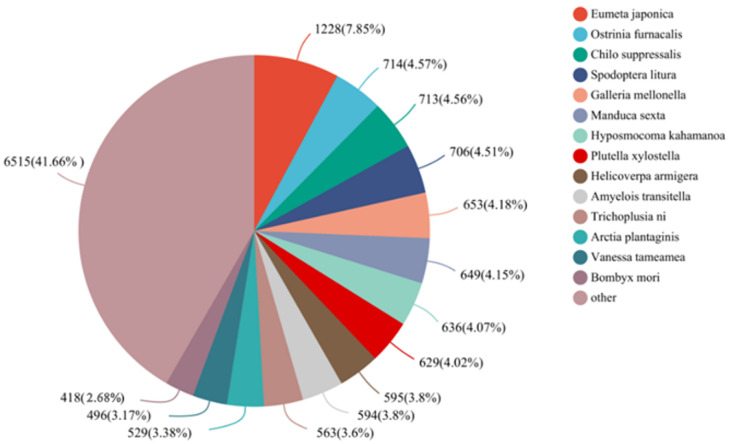
Species distribution of antennal unigenes of *E. signifer* in the Nr database.

Approximately 34.82% showed matches to entries in the Nr protein database through BLASTx, with an E-value cutoff of 1e-5. The top sequence matches were found in *Eumeta japonica* (1228, 7.85%), followed by *O. furnacalis* (714, 4.57%), *Chilo suppressalis* (713, 4.56%), *Spodoptera litura* (706, 4.51%,) and so on (Figure 2). 

**Figure 3:**
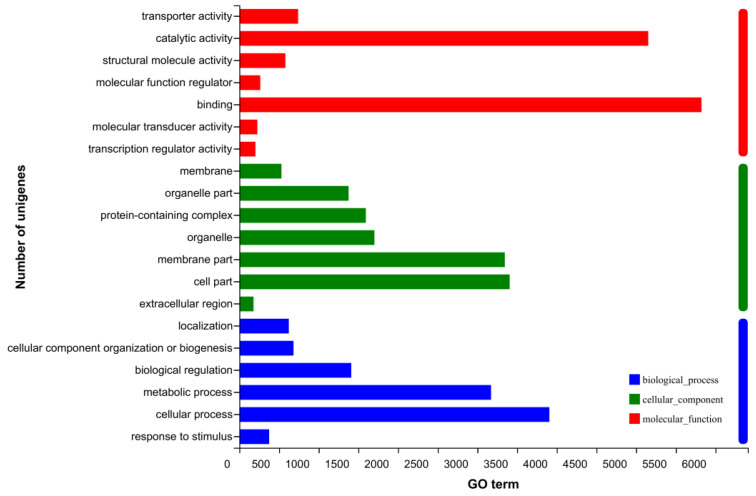
Gene ontology (GO) classification of assembled *E. signifer* unigenes.

The gene ontology (GO) annotations classified the 11,147 transcripts into various functional groups using BLAST2GO with P value calculated by a hypergeometric distribution test and E-value less than 1 × 10^–5^. Binding accounted for most of the GO annotations in molecular function (52.24%), cellular process accounted for most in biology process (35.05%), and cell part (30.56%) accounted for most in cellular component, in antennal transcriptome of *E. signifer* (Figure 3). 

**Figure 4:**
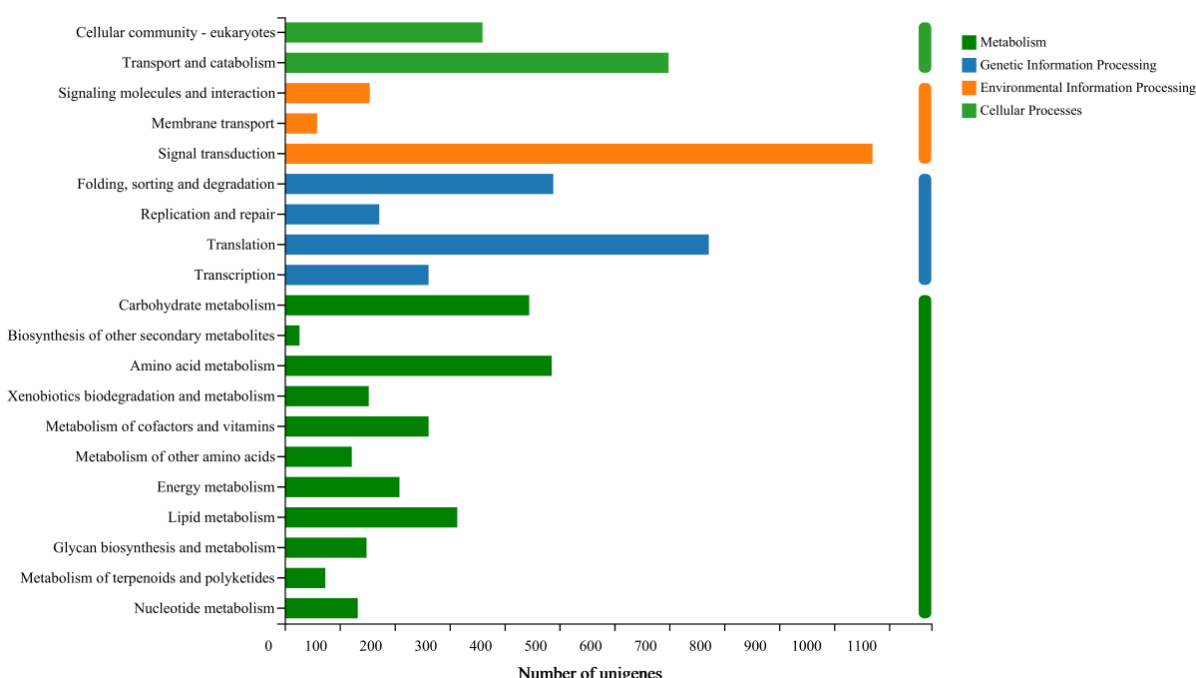
Classification of *E. signifer* Walker transcriptome based on KEGG.

Through the integration of KEGG database analysis, we identified 8929 unigenes participating in 4 metabolic pathways. Among these pathways, the top three most annotated ones are as follows: signal transduction accounting for 11.98%, translation representing 8.65%; transport and catabolism contributing to 7.83%, respectively (Figure 4).

### 2.3. Olfactory Proteins 

A total of 69 olfactory genes were identified. There are 22 transcripts encoding putative OBPs in *E. signifer* adult antennae, of which 5 were identified in the before head, thorax, and abdomen cuticula transcriptomes (Table 2 labeled with underline) [15]. Furthermore, among the identified OBPs, the top three in terms of expression levels were as follows: EsigPBP2 exhibited the highest expression, with a fragments per kilobase million (FPKM) value of 62,318.74; EsigPBP1 (also known as *EsigGOBP7*) followed closely behind, with an FPKM value of 53,518.17; *EsigGOBP13* ranked third in expression levels, with an FPKM value of 33,180 (Table 2). We identified 10 chemosensory proteins (CSPs), among which *EsigCSP1*, *EsigCSP2*, and *EsigCSP7* were previously identified (labeled with underline) [15], and the FPKM value showed *EsigCSP14*, *EsigCSP15*, and *EsigCSP10* were the top three genes in terms of expression levels (Table 2). A total of 19 ORs were identified in adult antennal transcriptome, in which only *EsigOR1* was identified before. It is interesting to observe that all *EsigORs* had much lower expression levels than binding proteins (*EsigOBPs* and *EsigCSPs*). Among *EsigORs, EsigOR4, EsigOR18*, and *EsigOR16* were most highly expressed (Table 2). We identified four transcripts encoding new gustatory receptor GRs. It is noteworthy that *EsigGRs* had much lower expression levels than most *EsigORs*, compared with *EsigORs* (Table 2). We identified 12 ionotropic receptors, IRs, among which known *EsigIR75p-6* was most highly expressed in the adult antennae (labeled with underline). Additionally, we successfully identified two new sensory neuron membrane proteins (SNMPs) in the antenna of *E. signifer* adult. All sequences of olfactory proteins were listed in Supplementary Materials Additional File S1.

### 2.4. Phylogenetic Analysis of OBPs and ORs

**Figure 5:**
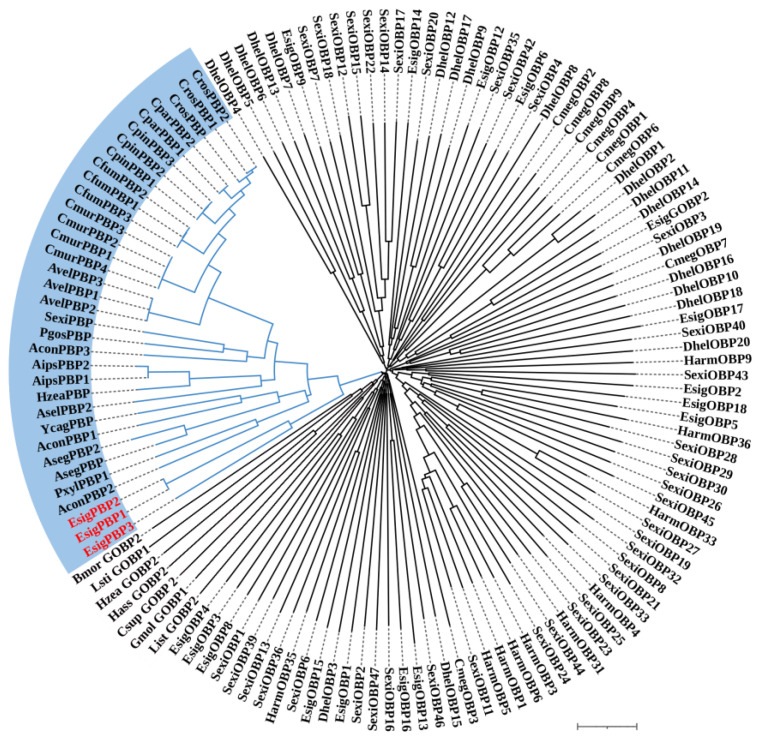
Neighbor-joining phylogenetic tree of odorant-binding proteins (OBPs).

**Figure 6:**
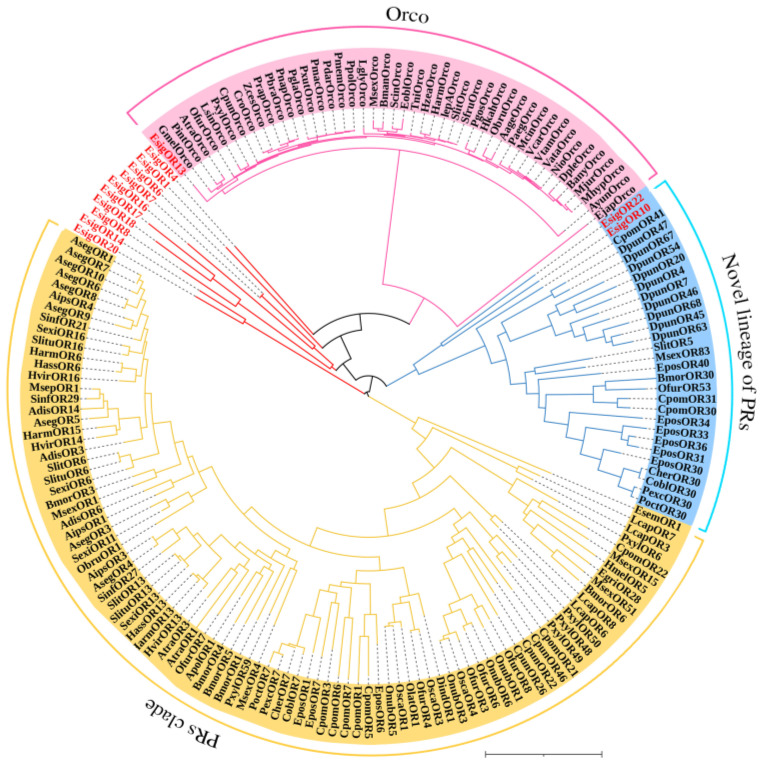
Neighbor-joining phylogenetic tree of odorant receptors (ORs).

According to the phylogenetic tree of OBPs supporting *EsigGOBP7* as the PBP of *E. signifer* [15], in this study we changed *EsigGOBP7* to *EsigPBP1*. In this phylogenetic tree of OBPs (Figure 5), the PBPs clades included *EsigPBP1* (EsigGOBP7, red), *EsigPBP2* (red)*, EsigPBP3* (red)*, PxylGOBP1*, and all Lepidoptera PBPs. The PBP clade had a 100% support rate (labeled with blue). In the NJ tree of ORs (Figure 6), we found there were three clades; the PRs clade (yellow) was found to be a sister clade to the clade that contained the novel lineage of PRs clade (blue), Orco clade (pink), and all other *EsigORs*. What is more, there is the close relationship between the clade of Orco and novel lineage of PRs. Additionally, *EsigOR10* and *EsigOR22* are in the clade of novel lineage of PRs, while *EsigOR13* was positioned within the Orco clade. Interestingly, there were no *EsigORs* found within the PRs clade itself (yellow).

The NJ phylogenetic analysis of OBPs of *E. signifer* was performed with reference OBPs of *Dastarcus helophoroides* [18], *Chrysomya megacephala* [19], *Plutella xylostella* [20], *S. exigua* [21,22], *H. armigera* [23], and PBPs of Lepidoptera. Blue branch was PBPs clade. The stability of the nodes was assessed by bootstrap analysis with 1000 replications. The scale bar represents 0.1 substitutions per site.

The NJ phylogenetic analysis of ORs of *E. signifer* (red) was performed with reference ORs of the novel lineage of candidate pheromone receptors and pheromone receptor clade sequence in Bastin-He’line et al. [24] and Orcos of moth. Blue branch was the novel lineage of PRs clade. Yellow branch was the PRs clade. Pink branch was the Orco clade. The stability of the nodes was assessed by bootstrap analysis with 1000 replications. The scale bar represents 1.0 substitutions per site.

### 2.5. Expression of Binding Proteins and Olfactory Receptors in Adult Antenna

**Figure 7:**
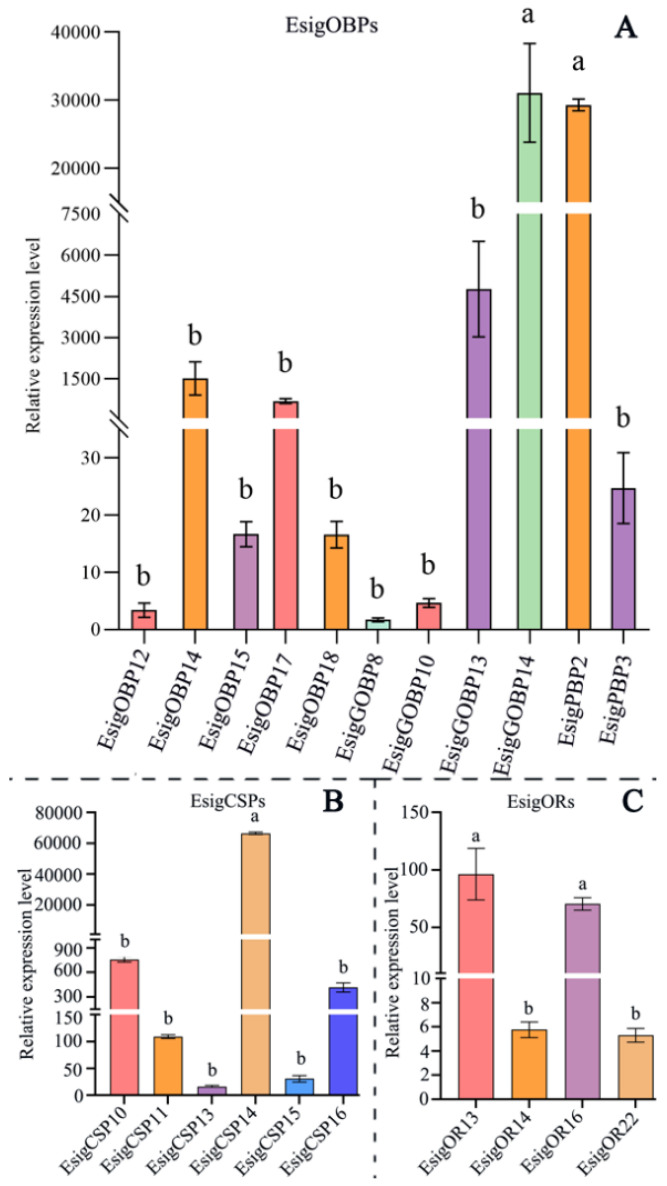
Expression of olfactory-related proteins in the antennae of *E. signifer* Walker. Note: (**A**) EsigOBPs, (**B**) EsigCSPs, (**C**) EsigORs. The error bar represents the standard error. The different small letters a, b on the error bar indicates significant differences at *p* < 0.05.

Based on the designed qRT-PCR primers and reference genes (Ribosomal protein, RIB, and Elongation factors, EF) [25]. The expression of 11 OBPs, 6 CSPs, and 4 ORs were determined in the adult antennae (Figure 7). All tested genes were expressed in adult antennae (Figure 7). For binding protein, *EsigCSP14*, *EsigPBP2*, and *EsigGOBP14* exhibited the highest levels of gene expression. Among OBPs, *EsigPBP2* and *EsigGOBP14* had significantly higher expression levels than other all OBPs (*p* < 0.05). For PBPs, *EsigPBP2* had significantly higher expression than *EsigPBP3* (*p* < 0.05). *EsigGOBP14* displayed significantly higher levels of expression compared to the other OBPs (*p* < 0.05), while the expression of the remaining OBPs did not exhibit any significant differences (Figure 7). In CSPs, the expression of *EsigCSP14* was significantly higher than that of the other five CSPs (*p* < 0.05) (Figure 7). In ORs, their expression levels were generally lower compared to those of the binding proteins. The expression levels of *EsigOR13* and *EsigOR16* were the highest among the odorant receptors, with no significant difference observed between them. Both were significantly different from *EsigOR14* and *EsigOR22* (*p* < 0.05) (Figure 7).

## 3. Discussion

In the adult antennal transcriptome of *E. signifer*, we identified 69 olfactory-related genes, including 22 OBPs, 10 CSPs, 19 ORs, 4 GRs, 12 IRs, and 2 SNMPs. Interestingly, in comparison to the larvae, the adult antennal transcriptome unveiled a significant increase, with the identification 17 new OBPs (including 2 pheromone binding proteins), 7 new CSPs, 17 new ORs, 4 new GRs, 11 new IRs, and 2 new SNMPs [15,16]. This notable expansion in olfactory proteins in adults can be attributed to their heightened need for olfactory capabilities in order to recognize a more complex chemical environment. Another reason is the size difference between the tiny larvae antennae and the adult antennae. The adult antennae are more specialized for olfaction, and therefore, the focus on olfactory proteins and the annotation of a greater number of olfactory proteins is naturally more pronounced. Interestingly, the consistent expression of *EsigGOBP2*, *EsigGOBP5*, *EsigCSP1*, and *EsigCSP2* across all *E. signifer* transcriptomes suggests that these proteins play a central role in olfaction throughout both the larval and adult stages. When comparing expression in olfactory tissues (larval head and adult antennae) from different development stages, *EsigGOBP2*, *EsigGOBP5*, *EsigCSP1*, *EsigCSP2*, *EsigCSP7*, *EsigOR4*, and *EsigIR75p-6* were consistently expressed among the third and twelfth larvae and adults, showing their crucial role in olfaction and implying their main olfactory function. Odorant receptors were found to be less common across different tissues and developmental stages in *E. signifer* [15,16]. All were similar to *Spodoptera littoralis*, in that the caterpillars expressed a smaller set of olfactory genes than the adults, with the exception of *SlitOBP21* and *SlitGOBP1*, which were adult-specific [26]. The identification of 69 olfactory-related genes in the antennal transcriptome of *E. signifer* adults places the species in the middle range when compared to other insect species. The number of olfactory-related genes is similar to that found in the antennal transcriptome of *Glenea cantor* [27], but fewer than in species like *Mythimna loreyi* with 138 olfactory-related genes [28], *Monochamus saltuarius* with 117 [29], and *Podabrus annulatus* with 101 [30]. On the other hand, *E. signifer* surpasses species like *O. loti* with 47 olfactory candidate genes in their antennae transcriptome [31] and *Reticulitermes aculabialis* with 16 olfactory genes [32]. 

Lepidoptera PBPs/GOBPs form a unique lineage of insect OBPs [33], and the number of PBPs varies among species [21]. The previous phylogenetic tree of OBPs supports *EsigGOBP7* as the PBP of *E. signifer* [15]; in this study, the PBPs clades also included *EsigPBP1, EsigPBP2, EsigPBP3, PxylGOBP1*, and all Lepidoptera PBPs, further supporting the theory that *EsigGOBP7* is a PBP. Gene expression analysis in PBPs revealed that *EsigPBP2* had significantly higher expression than *EsigPBP3* (*p* < 0.05), with FPKM values indicating a ten-thousand-fold higher expression level of *EsigPBP2* compared to *EsigPBP3*. This pattern is similar to the expression of PBP1 and PBP2 in *M. loreyi* [28]. Additionally, *EsigPBP1* also exhibited a ten-thousand-fold higher expression, suggesting that *EsigPBP1* and *EsigPBP3* have high expression tendencies and may serve as the main functional PBPs. Interestingly, in social insects, the expression of PBPs can exhibit role differentiation, with two PBPs showing significantly higher expression levels in alates compared to others in *R. aculabialis* [32]. For the newly identified OBPs, both qRT-PCR results and FPKM values supported *EsigGOBP14, EsigGOBP13, EsigOBP14*, and *EsigOBP17* as the fourth highest expression OBPs in adult antennae, suggesting their potential roles as key proteins in binding plant volatiles. This pattern is similar to *MusiOBP1*, which was the highest expressed in primordial females in *Megalurothrips usitatus* [34]. In addition, OBPs often exhibited a male antennae-biased expression pattern, as observed in examples like *OlotOBP1, OlotOBP4*, and *OlotOBP6* [31]. Moreover, OBPs are not restricted to expression in antennae; for instance, *GcanOBP22* and *GcanOBP25* were highly expressed in the wings and legs [27]. Furthermore, it is intriguing that the recognition between OBPs and host odors may be reduced by down-regulation in male *M. saltuarius* infested with *Bursaphelenchus xylophilus*, which could further affect the spread of *B. xylophilus* to new hosts [29]. Hence, it is crucial to explore the expression patterns of these key EsigOBPs in a more systematic manner to gain a better understanding of their roles in olfaction and host selection in *E. signifer*.

The results from both the FPKM values and qRT-PCR analysis confirm that EsigCSP14 exhibits higher expression compared to *EsigCSP10, EsigCSP11, EsigCSP13, EsigCSP15*, and *EsigCSP16*. The FPKM values indicate that *EsigCSP14* is 50- to 10,000-fold more highly expressed than other EsigCSPs, suggesting that *EsigCSP14* is the main and key CSP in adults and is not expressed in larvae. It is worth noting that the expression of CSPs can vary across different developmental stages; for example, *GcanCSP4* showed the highest expression in *G. cantor* female antennae at 12 days [27], and *MusiCSP1* was most highly expressed in *M. usitatus* primordial pupae [34]. Additionally, CSPs can be expressed in non-olfactory tissues and show division of labor biased in society insects. For example, five CSPs were more highly expressed in alates than in workers, soldiers, larvae, and nymphs, and the expression levels of *RacuCSP6* were significantly higher in *R. aculabialis* nymphs [32]. However, the developmental stages and tissue-specific expression patterns of *EsigCSP14* remain unknown, so further investigation is needed to reveal its detailed functions and roles in *E. signifer*.

It was found that ORs can be divided into two types: odorant receptorx (ORx) and Orco [35]. Functionally, Orco plays a crucial role in guiding the membrane targeting of canonical ORs [36]; moreover, it forms heteromerize with other ORs, through the conserved C-terminal and constituting ligand-gated ion channels, to become involved in the olfactory response of insect [37,38]. The homology of ORx among different insects was very low, and ORx was also varied among the same insects, which may be related to the recognition of odor substances in insect habitats [35]. However, Orco is a characteristic feature of olfactory sensory neurons (OSNs) expressing ORs and is highly conserved across various insect species and orders [35]. The phylogenetic evolution of insect ORs supported that the insect olfactory system has expanded its receptor repertoire with the occurrence of Orco proteins in Zygentoma, and the common origin of all insect Orco was Microcoryphia Orco [39]. In the case of *E. signifer*, all Lepidoptera Orco proteins formed a distinct cluster, and *EsigOR13* was classified within the Orco clade, suggesting that it serves as the Orco receptor. Both the qRT-PCR results and FPKM values indicated that *EsigOR16* exhibits the highest expression among the odorant receptors, making it the key receptor involved in odorant recognition. Likely, in *O. furnacalis*, *OfurOR8, OfurOR7*, and *OfurOR5b* primarily respond to the sex pheromone components of other *Ostrinia* species, while *OfurOR27* strongly responds to plant odorants such as nonanal, octanal, and 1-octanol [9]. These findings highlight the specificity and diversity of odorant receptors in different insect species, reflecting the species’ adaptations to specific ecological niches and behaviors.

In the phylogenetic tree analysis, several interesting relationships among odorant receptors (ORs) in *E. signifer* were observed: (1) Relationship between the PRs clade and the novel lineage of PRs clade: The PRs clade was found to be the sister clade to the clade containing the novel lineage of PRs clade, the Orco clade, and all other EsigORs. This suggests a close relationship between the Orco clade and the novel lineage of PRs clade, both of which are distinct from the traditional PRs clade. (2) Absence of EsigORs in the traditional PRs clade: In Lepidoptera insects, there is a conserved clade of ORs that is specialized in sensing female sex pheromones and, thus, is called the traditional PRs clade [40], such as the five MlorPRs in this clad (*PR1, PR2, PR3, OR1*, and *OR14*), and each of the *MlorPR* is closely grouped with one or more PRs from other moths [28]. But, in our result, no EsigORs were found in this traditional PRs clade in *E. signifer*. (3) Novel PRs clade: Recent research has identified a new PR clade in Lepidoptera insects that is more closely related to general ORs [24]. This new clade was also observed in *E. signifer*, with *EsigOR10* and *EsigOR22* grouped within this novel PRs lineage. This suggests that these receptors may have functions related to sensing sex pheromones, similar to *SlitOR5* in other moths [24]. (4) Evolutionary implications: The absence of traditional PRs in *E. signifer* and the closer relationship between the novel PRs lineage and Orco in the phylogenetic tree raise interesting evolutionary questions. This is combined with the view that the insect olfactory system has expanded its receptor repertoire with the occurrence of Orco proteins in Zygentoma [39] and ended up with the versatile OR complexes in flying insects [39,41] and the primitive moth *E. signifer*. It is possible that the novel PRs lineage represents a more primitive form of pheromone receptors in moths, and the evolution of specialized PRs may have occurred in a lineage-specific manner.

## 4. Materials and Methods

### 4.1. Collect Insect and Tissue 

The *E. signifer* larvae were collected from a damaged eucalyptus plantation between December 2019 and April 2022 at the Gaofeng forest station (N 22.907°, E 108.266°), Guangxi, China. Subsequently, the larvae were artificial fed to adulthood, and their antennae were cut and collected for further analysis.

### 4.2. Construct cDNA Library and Sequence

The total adult antennal RNA was extracted by using a TRIzol reagent (Ambion, Naugatuck, UK) and the RNeasy Plus Mini Kit (No. 74134; Qiagen, Hilden, Germany); then, both density and quality were examined. Three cDNA library construction and Illumina sequencing (HiSeq2500 platform) of three separate RNA samples were performed at MajorBio Corporation (Shanghai, China), respectively. The entire cDNA library preparation process, including mRNA sample purification, fragmentation, first-strand cDNA synthesis, end repair, and PCR amplification, followed the methodology outlined by Zhang [15]. 

### 4.3. Assembly, Functional Annotation, and Olfactory Genes Identification 

The raw reads acquisition, clean read assembly and evaluation were performed as per Zhang [15] and used BUSCO to evaluate the assembly integrity score—the higher the score, the better the integrity. NCBI BLASTx searches were used to annotate unigenes and the identification of olfactory proteins (OBP, CSP, OR, GR, IR, and SNMP) was checked by tBLASTn manually. The Blast2GO pipeline was used to perform GO annotation. The FPKM values (fragments per kilobase per million reads) were used to represent the genes’ expression levels [42], which were calculated by RSEM (RNA-Seq by Expectation-Maximization) (Version: 1.3.1) with default parameters [43]. 

### 4.4. Sequence and Phylogenetic Analysis

Muscle was used to align amino acid sequences, then the Mega v6.0 software package [44] was used to construct a neighbor-joining (NJ) tree [45] of OBPs with a P-distance model and a pairwise deletion of gaps. Color and arrangement of the NJ tree used FigTree (Version 1.4.2). The reliability of the tree structure and node support was evaluated by bootstrap analysis with 1000 replicates. Considering that *E. signifer* is a primitive Lepidoptera moth, the phylogenetic analyses of the OBPs were based on PBPs of Lepidoptera and OBPs of *Dastarcus helophoroides* (Coleoptera) [18], *Chrysomya megacephala* (Diptera) [19], *Plutella xylostella* [20], *S. exigua* [21,22], *H. armigera* [23] in Lepidoptera, and all OBPs of *E. signifer* [15,16], including those formerly and newly identified in larvae and adult antennal transcriptomes. The ORs tree used all Lepidoptera Orco, all ORs of *E. signifer* [15,16], and the novel lineage of PRs and PR clade sequence in Figure 4 of Bastin-He’line et al. [24]. The gene names and GenBank numbers of *P. xylostella*, *H. armigera*,Lepidoptera PBPs, and ORs in Bastin-He’line et al. [24] are listed in Supplementary Materials Additional File S2, and the other gene sequences are listed in the reference articles. 

### 4.5. Expression Pattern of Olfactory Proteins in Adult Antennae

Expression patterns of 11 OBPs, 6 CSPs, and 4 ORs in adult antennae were constructed. The method used to extract the RNA of the adult antennae and test the quality was carried out as described before. cDNA was synthesized with the TransScript One-Step gDNA Removal and Synthesis Super Mix (No. O10306; Trans, Beijing, China). Primers were designed using Primer3 http://bioinfo.ut.ee/primer3-0.4.0/ (accessed on 15 December 2022) (Table 3), and the reference genes were determined as per Chen [25]. The PCR analysis was conducted using a Roche LIGHT CYCLE 480II (Colombia, SC, USA). Genious 2X SYBR Green Fast qPCR Mix (No ROX) (No. RK21205; ABclonal, Wuhan, China) was used for the PCR under a three-step amplification. Each PCR was conducted in a 20 µL reaction mixture containing 10 µL of Genious 2X SYBR Green Fast qPCR Mix (No ROX), 0.8 µL of each primer (10 mM), 2 µL of sample cDNA (2.5 ng of RNA), and 7.2 µL of dH_2_O (sterile distilled water). The qRT-PCR cycling parameters were as follows: 95 °C for 180 s, followed by 40 cycles of 95 °C for 5 s, 60 °C for 30 s, and 65 °C to 95 °C in increments of 0.5 °C for 5 s to generate the melting curves. The negative control was performed without either template. Each gene analysis was performed in three biological replicates and three technical replicates. Roche LIGHT CYCLE 480II was used to normalize the expression based on ΔΔCq values, using the reference genes EF and RIB, and using *EsigGOBP8* and *EsigOBP12* as control samples, and the 2^−ΔΔCt^ method was used [46]. The normal distribution and equal variances test were performed and all the logarithm data that followed a normal distribution with equal variances were examined before comparative analyses. The comparative analyses for each gene were assessed by a one-way nested analysis of variance (ANOVA), followed by Tukey’s honestly significance difference (HSD) tests implemented in SPSS Statistics 18.0. The values are presented as the means ± SE.

## 5. Conclusions

The analysis of the antennal transcriptome in adult *E. signifer* revealed a total of 69 olfactory-related genes, which included 22 OBPs, 10 CSPs, 19 ORs, 4 GRs, 12 IRs, and 2 SNMPs. When compared to the larval transcriptomes, this adult antennal transcriptome uncovered 17 new OBPs, comprising 2 PBPs, 7 CSPs, 17 ORs, 4 GRs, 11 IRs, and 2 SNMPs. Through the construction of phylogenetic trees for OBPs and ORs, three PBPs, one Orco, and two new PRs of *E. signifer* were identified. An in-depth analysis of the relationship between the novel PRs clade and Orco, the evolution of Orco and ORs, and the characteristics of the primitive moth *E. signifer* led to the hypothesis that the novel lineage of PRs clade may represent primitive PRs in moths.

## Figures and Tables

**Table 1:** Quality control, number, and length of unigenes in *Endoclita signifer* adult antennal transcriptome.

Quality Index	Adult Antenna		
	Duplication 1	Duplication 2	Duplication 3
Raw reads	50,604,462	44,113,510	49,462,752
Clean reads	50,001,732	43,609,530	48,952,368
Q20 (%)	98.2	98.05	98.19
Q30 (%)	94.45	94.09	94.43
GC content (%)	43.81	43.73	43.98
Total transcripts number	62,439		
Total unigenes number	44,905		
Largest unigenes length (bp)	17,261		
Average unigenes length (bp)	859.46		
N50 of unigenes	1488		
BUSCO of unigenes	C: 89.30% [S: 85.7%; D: 3.6%]		

**Table 2:** Best BLASTx hits for putative chemosensory proteins of *Endoclita signifer*.

Name	Nr Description	Species	Acc. NO.	FPKM3	FPKM2	FPKM1	Average FPKM
EsigOBP6	odorant-binding protein 16	*Ectropis obliqua*	ALS03864.1	6300.48	6312.6	6477.34	6363.47
EsigOBP11	circadian clock-controlled protein-like	*Papilio xuthus*	XP_013175982.1	1.74	3.07	2.41	2.41
EsigOBP12	putative odorant-binding protein 8	*Conopomorpha sinensis*	QGN03642.1	318.63	293.03	313.13	308.26
EsigOBP13	minus-C odorant-binding protein 3	*Batocera horsfieldi*	ADD82416.1	2.1	2.81	6.27	3.73
EsigOBP14	putative odorant-binding protein 8	*Conopomorpha sinensis*	QGN03642.1	3141.39	3101.9	3243.38	3162.22
EsigOBP15	putative odorant-binding protein 3	*Conopomorpha sinensis*	QGH51239.1	115.86	115.33	123.33	118.17
EsigOBP16	odorant-binding protein 2	*Monochamus alternatus*	AHA39267.1	3.05	5.05	4.72	4.27
EsigOBP17	Pheromone-binding protein female 1, partial	*Loxostege sticticalis*	ACF48467.1	1064.72	1026.4	1075.55	1055.56
EsigOBP18	pheromone-binding protein 2-like	*Amyelois transitella*	XP_013191569.1	3.11	1.43	0.61	1.72
EsigGOBP2	general odorant-binding protein 56d-like	*Hyposmocoma kahamanoa*	XP_026319368.1	4.52	10.11	11.49	8.71
EsigGOBP3	general odorant-binding protein 83a-like	*Plutella xylostella*	XP_011554700.1	1376.12	1368.2	1442.07	1395.47
EsigGOBP5	General odorant-binding protein 19d	*Eumeta japonica*	GBP31818.1	9.00	7.64	8.17	8.27
EsigGOBP8	general odorant-binding protein 19d-like	*Papilio xuthus*	XP_013173035.1	1.86	1.24	2.18	1.76
EsigGOBP9	general odorant-binding protein 99a	*Danaus plexippus plexippus*	XP_032518123.1	1.04	2.99	0.00	1.34
EsigGOBP10	General odorant-binding protein 67	*Eumeta japonica*	GBP19217.1	16.15	14.28	8.92	13.12
EsigGOBP11	general odorant-binding protein 70-like	*Amyelois transitella*	XP_013201142.1	4.61	4.43	4.24	4.43
EsigGOBP12	general odorant-binding protein 56a-like	*Plutella xylostella*	XP_011557121.1	22.52	0.00	3.07	8.53
EsigGOBP13	general odorant-binding protein 2	*Sitotroga cerealella*	AII15785.1	32,192	33,991	33,357.26	33,180.01
EsigGOBP14	General odorant-binding protein 2	*Epiphyas postvittana*	Q95VP2.1	6823.62	6786.8	7338.43	6982.95
EsigPBP1	general odorant-binding protein 1	*Athetis dissimilis*	ALJ93806.1	52,549.2	53,582	54,423.05	53,518.17
EsigPBP2	general odorant-binding protein 2	*Sitotroga cerealella*	AII15785.1	61,670.1	61,939	63,346.96	62,318.74
EsigPBP3	general odorant-binding protein 1	*Dendrolimus kikuchii*	AGJ83357.1	2583.86	2496	2685.59	2588.47
EsigCSP1	chemosensory protein 10	*Carposina sasakii*	AYD42214.1	415.55	437.59	441.02	431.39
EsigCSP2	chemosensory protein 24	*Cnaphalocrocis medinalis*	ALT31606.1	4.15	4.76	7.52	5.48
EsigCSP7	chemosensory protein	*Eogystia hippophaecolus*	AOG12893.1	5.18	2.48	3.33	3.66
EsigCSP10	chemosensory protein 1	*Dastarcus helophoroides*	AIX97069.1	1146.33	1146.3	1189.32	1160.63
EsigCSP11	chemosensory protein 1	*Mythimna separata*	AWT22249.1	74.89	74.72	72.82	74.14
EsigCSP12	chemosensory protein 13	*Mythimna separata*	AWT22251.1	17.81	19.75	18.34	18.63
EsigCSP13	chemosensory protein CSP23	*Lobesia botrana*	AXF48719.1	118.42	89.29	98.44	102.05
EsigCSP14	ejaculatory bulb-specific protein 3-like	*Trichoplusia ni*	XP_026729747.1	26,772	26,128	27,166.52	26,688.84
EsigCSP15	ejaculatory bulb-specific protein 3-like	*Amyelois transitella*	XP_013187502.1	4680.67	4567.5	4813.77	4687.31
EsigCSP16	microsomal glutathione S-transferase 1-like	*Myzus persicae*	XP_022165210.1	57.5	58.91	60.89	59.10
EsigOR1	odorant receptor Or1-like	*Anoplophora glabripennis*	XP_023310030.1	7.00	8.87	6.95	7.61
EsigOR4	odorant receptor OR3	*Rhyacophila nubila*	AYN64393.1	41.55	42.52	46.63	43.57
EsigOR6	putative odorant receptor 85d	*Drosophila sechellia*	XP_002032031.1	0.29	0.22	1.17	0.56
EsigOR7	odorant receptor 4-like	*Ctenocephalides felis*	XP_026480036.1	1.86	0.53	2.35	1.58
EsigOR8	odorant receptor OR15	*Colaphellus bowringi*	ALR72560.1	2.77	2.87	1.92	2.52
EsigOR9	gustatory and odorant receptor 22-like	*Amyelois transitella*	XP_013186820.1	9.56	8.23	2.29	6.69
EsigOR10	odorant receptor OR4	*Rhyacophila nubila*	AYN64394.1	2.45	1.78	2.90	2.38
EsigOR11	odorant receptor 85c-like	*Danaus plexippus plexippus*	XP_032521521.1	1.71	1.31	2.86	1.96
EsigOR12	odorant receptor 27	*Conogethes punctiferalis*	ARO76432.1	14.88	10.11	19.5	14.83
EsigOR13	odorant receptor co-receptor	*Eriocrania semipurpurella*	ATV96621.1	3.18	2.92	4.24	3.45
EsigOR14	odorant receptor 47a-like	*Nylanderia fulva*	XP_029159982.1	12.11	15.01	14.2	13.77
EsigOR15	odorant receptor 59b-like	*Drosophila serrata*	XP_020801244.1	2.39	2.07	1.75	2.07
EsigOR16	odorant receptor 13a	*Ctenocephalides felis*	XP_026480046.1	27.52	26.33	30.39	28.08
EsigOR17	odorant receptor 13a-like	*Hyposmocoma kahamanoa*	XP_026322472.1	5.42	4.13	4.53	4.69
EsigOR18	odorant receptor 49a-like	*Papilio xuthus*	XP_013164627.1	40.63	39.96	37.37	39.32
EsigOR19	odorant receptor 49b-like	*Vanessa tameamea*	XP_026496790.1	1.29	1.48	0.00	0.92
EsigOR20	odorant receptor 22c-like	*Temnothorax curvispinosus*	XP_024869954.1	1.04	1.19	1.63	1.29
EsigOR21	odorant receptor	*Eogystia hippophaecolus*	AOG12928.1	0.50	2.31	0.50	1.10
EsigOR22	odorant receptor OR4	*Rhyacophila nubila*	AYN64394.1	9.64	4.05	3.18	5.62
EsigGR4	gustatory and odorant receptor 22-like	*Amyelois transitella*	XP_013186820.1	9.56	8.23	2.29	6.69
EsigGR5	gustatory receptor for sugar taste 43a	*Zeugodacus cucurbitae*	XP_011189783.1	1.22	0.00	1.84	1.02
EsigGR6	putative gustatory receptor GR55, partial	*Hedya nubiferana*	AST36215.1	0.37	4.24	0.74	1.78
EsigGR7	gustatory receptor for sugar taste 43a-like	*Pieris rapae*	XP_022122807.1	2.58	2.12	4.82	3.17
EsigIR25a-2	ionotropic receptor 25a	*Bombyx mandarina*	XP_028034019.1	2.77	1.22	1.07	1.69
EsigIR13	ionotropic receptor, partial	*Glyphodes pyloalis*	QIJ45776.1	0.00	0.00	0.00	0.00
EsigIR18	putative ionotropic receptor IR7d.2, partial	*Athetis lepigone*	AOE47993.1	11.75	11.1	12.41	11.75
EsigIR75p-8	putative ionotropic receptor IR75p.1	*Hedya nubiferana*	AST36233.1	0.89	1.28	0.90	1.02
EsigIR19	ionotropic receptor 7d1, partial	*Heliconius telesiphe sotericus*	AMM70701.1	2.01	1.54	1.42	1.66
EsigIR93a-6	ionotropic receptor 93a	*Conogethes pinicolalis*	QEE82793.1	1.62	1.24	1.27	1.38
EsigIR75p-6	putative ionotropic receptor IR75p.1	*Hedya nubiferana*	AST36233.1	36.48	29.57	27.55	31.20
EsigIR75a	ionotropic receptor 75a-like	*Hyposmocoma kahamanoa*	XP_026318656.1	0.00	0.00	1.02	0.34
EsigIR14	ionotropic receptor IR13	*Lobesia botrana*	AXF48844.1	22.32	23.67	22.94	22.98
EsigIR15	putative ionotropic receptor 9	*Conopomorpha sinensis*	AXY83439.1	3.41	0.78	3.35	2.51
EsigIR16	glutamate receptor ionotropic, delta-1	*Plutella xylostella*	XP_011560609.1	11.78	11.58	11.11	11.49
EsigIR17	ionotropic receptor 93a-like	*Ostrinia furnacalis*	XP_028174055.1	5.66	5.12	6.19	5.66
EsigSNMP2	sensory neuron membrane protein	*Dioryctria abietella*	QJX59445.1	2.38	2	2.57	2.32
EsigSNMP3	putative sensory neuron membrane protein 2	*Ectropis obliqua*	ANA75033.1	16.12	18.84	17.89	17.62

Note: olfactory proteins with underline had been identified before [15].

**Table 3:** Primers for real-time fluorescent quantitative PCR.

Genes	Forward Primer	Reverse Primer
EsigOBP12	CCAGTCAATCTTTTCTGGAC	TGCAAAGCTTTATCATCTCA
EsigOBP14	CGAAGAGATAACTGGCGTAG	GAAGTGGATACAGTCCTTGC
EsigOBP15	ATGCTTTTGCATACGGTTAT	TCGCCATTAGATGTTTCTTT
EsigOBP17	GGAGATGATGACAACCCTAA	CCATAATTCCTGTTCGTTGT
EsigOBP18	TACAAGACTGTAGGCATTCTG	CTGTTCTCGTTGAGCATACA
EsigGOBP8	CAGTCTCTGACGAGGAAGTC	CTGTTAGCGACCTTCATACC
EsigGOBP10	CATGGAGGAAATTAAGGGCT	TAACACGAGGACTTTACGTG
EsigGOBP13	CTAACACCGGAAATAATGGA	TGATGAAATCATCCATGTTC
EsigGOBP14	GGAAGAGTTCCTCCACTTCT	TGGTGGAAGGATTTGATG
EsigPBP2	AAAGCCAGATGGACTTATCA	ATCGAACTTCTTGTTCATGC
EsigPBP3	GGATGTAACTATTGGCTTCG	AACTTCTTCTCGCAGTTGTC
EsigCSP10	GCAACGAGTTGAAGAAGAAC	AGCTCTGCGCTCTTTAAGTA
EsigCSP11	ACAATACCAGTTTCCACCAC	TGGGATCCTAATTGATGAAG
EsigCSP13	CACGAAATGCAATCCGAAG	TTTACGCATAAATCAATGGCT
EsigCSP14	TGTAGATCACCTTGAAGACG	CGGTCTTCGTACTTCTTCCT
EsigCSP15	GACTACGACGTGGACAGTTA	TTCTCGTACTGCTCCTTCTG
EsigCSP16	GCTAAAACTCCAAAAGCAAA	GTTGGTCGCTAGGAATACTG
EsigOR13	ACATACGGCACAGCTCTACT	TTCAATGAGTGTGTTTCCAA
EsigOR14	TTCTTCTATTCCTGCTTTGC	ACTCGCAATGTCTTCTTGTT
EsigOR16	TTGTCTACATGAGCAACTGG	GAATGTGCGTAGAATTGTCA
EsigOR22	CGTGGTATGGAGACAAAGTT	AGATTGATGTCGCTGAAAAT

## Data Availability

Data are contained within the article and Supplementary Materials.

## Supplementary Materials

The following supporting information can be downloaded at: https://www.mdpi.com/article/10.3390/life13122264/s1, Additional File S1 Nucleic acid sequences of all candidates’ olfactory proteins identified in *Endoclita signifer* antennal transcriptome. Additional File S2 The protein names and gene accession numbers were used in phylogenetic trees.
