# Supplementary material for: A Study of Adult Olfactory Proteins of Primitive Ghost Moth, Endoclita signifer (Lepidoptera, Hepialidae)
Source: Life (Basel). 2023 Nov 27;13(12):2264. doi: 10.3390/life13122264 (PMC10744962; doi:10.3390/life13122264)
Supplement: Supplementary file 1 [file life-13-02264-s001.zip › Supplementary file S1.pdf]

A Study of Adult Olfactory Proteins of Primitive Ghost Moth, *Endoclita signifer* (Lepidoptera, Hepialidae)

Guipeng Xiao [1], Jintao Lu [2], Zhende Yang [2], Hengfei Fu [2,*] and Ping Hu [2,*]

Additional file S1 Nucleic acid sequences of all candidates' olfactory proteins identified in *Endoclita signifer* antennal transcriptome

EsigOBP11

ATTCTTATTTTACAAATTGCAGGCGACTTGGCCAACGACCTTGGCAATAACAATTGGAGA
CTGCTCTACCGGGAGATGCTGCCCGTCGCTCAACGCAATTGGGATAAGAGCGGCAAAAGA
GTTGGCCACAAACTGTTTCTGAAGGTTCCCTACAACCAACTGTTCCCCACGTCGGGTTAA

EsigOBP12

ATGTGGAACTATATGTTGGTTTTGGTGGTGGTCGCTCGAGCTGAAGTGGAATGGCCCAGT
CAATCTTTTCTGGACCAGATGCAGCCTATTCTAGAAAAGTGCTTATCGGAAAATCCAGTA
GACGCAGGTGTTTTAGAAAAAGCAAAAGCAGGCGATTTGGGCGGCGACCCAGATTTGAAG
AAACTTTTGCTATGCAATTTCGACATGGAAGGAGTGATCGACCACGATACTGGAGAGATA
TTTTACTACAAACTGCTGGACATGCTGCCAAACCAGGAGACCAAACGGCACGTGTTTGAG
ATGATAAAGCTTTGCATCGGCATACACAACCCGGCAGCGCAGGACACGGCGCTGCAATTC
GCCAGATGCATGAGGGACAAAGGCAGTCCTTTCTTCTTTATCTAA

EsigOBP13

ATGAAGATTGTTATCGTGATGTTGTGTACCATTGTGGGCATTTGGGCCCAAGACAAGAAG
CTCATCGCCGAGGAGCTGATGTTGGAGCACATCCACGACGAGTGCCAAGCCGACCCGGCC
ACCTACGCCGACCACGAGCTCCTCCACCACCTCTCAGAGAACATCGACAACCCCCAAGTA
GGCGCCCACATGCTCTGCGAATCTACCAAGGTAGGACTGCAGAAGGAGAACGGGGAATTG
GATATCCCCACCATCAAGGAGAAGATCGGCCTTACAGTCCAGGATCCCGACAGAGTGGAG
TTCCTGGTGAAGGAATGCGCCATTAAGAAGAACACTCCGGAGAAGACCGCCATCAATCTC
TTC

EsigOBP14

ATGTTTAAGGGCATAATCTATATTTTGGCAGGTTGTCTCGTTTTTGCGAGTATTAGTGGA
GAAGAGTTTCCTACTCAAGAACTGATCGACATGTTGGCACCAGTGGTGAGCAAATGCGAA
GAGATAACTGGCGTAGACAAAGATCTCGTTACTCATTTCTCCGAGGGTCGCATGCCGGAT
GACCCCGCACTGAAGTGTTACATGAAGTGTATTTTTGTGGAATTCGAAGTGCTCGACATT
CAAACTGGAACATTTCACTACGAGAAGATGCTGGATGCGATACCGGAATCCATGAAAAAG
ATAGCCTATAACATGGGCAAGGACTGTATCCACTTCAAGGGCGAGGGAGGCAGTGACCTC
TGTCAAGTGTCTTATGATCTTCATAAATGTTGGCAACAGGCTGATCCTGAACATTATTTC
TTACTGTAG

EsigOBP15

**ATGCTTTTGCATACGGTTATATCTTTTGTGCTTATATTTTCGACTAACGCTCTGAAATGC
AACACGGATAAAGGACCAAACGAAGAAGAAATGAAAAGTGTATTAATGAATTGCATGAGG
AAACTTGGGCTTCCACTACCTGGACAACTTGATGTGCTAGGAGATGGCAATTTTTCTATG
GAATATAATAAAGAAACATCTAATGGCGAAATTTCCAGTAATAAAGGCCACTCATTGAAT
TACAGAAGAGCTGTCATACCTTCTCACAGACGTTACAACCGTGACACAAGCCATGGAACT
ATGACGACAAACACAGGACAGAGGAGCCAATATAATCCAAACATAGCAACTAGATTTGAT
CACGACAGTTCAGGCAGTGAAGAATCCGATTACGGTACTAATAATCAAGATTTAAATGAC
AGCAATCGTACCGAATTCATAAATAACAATTCATATGATGTACTACGAAAAAAGGCCTGT
GTCACACATTGCTTTCTGCAGGACTTTCAAATGACCAGTGAGAATGAAATGCCTGACAGG
CATCTGATGGCAAACTTTATGACTCGAGACATGAGCAATAAAGAATATAAAGATTTTGTA
AATGAATCCATTGAAGACTGTTTTAAACTTATACAAATAGAAAATATCACGGACAAATGT
GAATTCTCCAAGAATATCTTCAAATGTATTTCAGAGAAAGGTCGTGCAAATTGCGACGAT
TGGAGTGAACGTATTTCCTTTTTTTAA**

EsigOBP16

**ATGAAGGCTGTTGTTGTTTTTTTGTGTGCCATCGTCGCAGGAGTTTTGGCTCAACTTCCT
GACAGCGAACGGGTATTCCTGAAACAAGTCCACGATTCCTGCCAAGCCGACCGCGCCACC
TACGCCGACGAGAGCCGCCTGAAGCAGCTCAGCAAGTACATCGACGACCCCGTCATAGGC
AAACATATGCTGTGCATGTCCCAGAAGGCGGGTCTTCAGAAGGGGAATGGCGACCTGGAC
ATCGCCGTTATCAAAAAGAAGATCGACCTCGTGACTCCTGACAAGTCCAAGGTGGACGGT
TTGGTCAAGAAGTGCGCCGTTGTCAACGGGAATCCCGGGAGGACCGCCAACTTGCTCTGG
CTGTGTTTC**

EsigGOBP8

**ATGAAGTTAATTGTGCAATGCCTCGCGTATATTCTTCTAGTGATATGCCACAGCGCATTA
GCAGTCTCTGACGAGGAAGTCAAGGCTAAATTCATGCAAACAGTACTCGAATGTATGAAA
AAGACGCCGCTTACGCCAGACGATATGACTTCGTTGAAAAATCATATCATACCAGAGACG
GGAAATGCCAAGTGCGTTTTGGCCTGCGCCTATAAAAGCATAGGTGTCATCGACGAAAAG
GGATTGTTTACTGGAGAAGGAGGTATGAAGGTCGCTAACAGATTATTCCCGGGAGACGAC
GAGAAATTGAAGAAATCTAAGGAATTTGTAAACATTTGCAGCACAATAAATAACGAACCA
GTGTCAGACGGTGTAAGCGGATGTGATCGTGCTGGCCTACTGTTCAAATGTGTCAGGATA
AATGGACCCAAACTTGGATTCCGAATTTGA**

EsigGOBP9

**ATGACTGGAGTACAATTGTTGTTCGCTGCCTGTATGGTGCTCGTGGGATCGGTTCAGTGC
TACTCCGAGGACGAAGTAAAAGCGAAGGGTACAGAGCTATTTCTGCAGTGTGCAGAAGGT
CAGACCGTGGCAAAGGAAGACTTAGAAAAACTGGAGAAACAGGAGTTACCGGAAAATGAT
AACGTTAAGTGTATATTGGCCTGTCTTTACAGAAAATTCAATGCTATCGATGACCAAGGC
TTATTCACGCCAGAAAAGGAATCGGAAACTGGACGGGAACTATACGCCGGTGCCCCT**

EsigGOBP10

ATGTTTACCGTTGTTTCGATAAATTTATCTATACTTCTCATATTGACGGTCTTGGAAAAA

AATGTTGCACTAGGCTATACATGTGGCTCACCTCCTAGTATGAACATGCAGATCTGTTGC
AATGGATTAAATTTTGTAGATAAAGCCATTAAGGAGGAGTGCATGGAGGAAATTAAGGGC
TCACCGGCTTCAGAAATATACTGCAATAAAATCAACTGCTTGCTTAAAAAAAAACGCTTT
GTACAAGATGACGGAGCAGTAGACTTACCAGCAATAACTGCACACGTGAGCAAGTGGGTC
GGGGAGAATCCCGAATGGTCGACCTTGCCAGCGGTAATCAACGACGAATGTTTCACAGAC
AAATTATCGCAGCTAGAATTTAAGCCCAGCTGTCAAAAAAACAAGGTGTTTTACTGTATC
GCACGTAAAGTCCTCGTGTTATGTCCCGCTAGCCGCTGGACTGATTCCGCTGAATGTAAT
GAGATTAAGAAGTACTTGGAGGAGTGCAAATAA

EsigOBP11

ATGGTTCGGAAAAGTAGTGTGATATTGTGTTTTTTGGTGTGTGTTAATTTTGGAATCTCG
TTGTCTAGCAGCGCTATATCATCGGAAAGCGAAAAGCGATGCAGGAACCCGCCGACGGCG
CCGCAGAAGATCGAGCGGGTGATTACGCAGTGTCAAGATGAAATTAAAATATCCATCCTT
CGAGAGGCGCTGGACGTCATAAAGGAGGAGCACACGATGCCGCAGAAGCGGACGCGCAAC
AAGAGGGAGGTGCCCTTCACGCACGACGAGAAGAGGATTGCTGGATGCCTGCTGCAGTGC
GTCTACAGGAAAGTGAAAGCGGTTGACGGGTACGGCTTCCCGACGCTCGAGGGCCTGGTG
CGGCTCTACTCGGACGGCGTGAACGAGCGCGGCTACTTCATGGCGACGCTCGAGGCTAGC
CGCGATTGCCTCATGAGGAACCACGACAAGTTCTCCAGGACCGTTCCCATGGATAACGGA
CGCAACTGCGACGTGTCCTTCGACATTTTCGAGTGCATCTCGGACAGGATCGGCGAATAC
TGCGGCAACAACGGCCTGTGA

EsigGOBP12

ATGTTTTGTCTGGTTTTCGTGGCTGGTTTGAATTTGGTGCTTTCAAAGCCAGATGGACTT
ATCATGAAAACTATCAGCGTTGGCTTCGGAAAAGCTTTGGATGAATGCCGTGAGGAGAGT
AATCTAACACCGGAAATAATGGAAGAGTTCCTCCACTTCTGGAGGGACGACTTTGAGATC
AAGCACCGGGAGCTGGGTTGCGCCATCCTCTGCATGAACAAGAAGTTCGATCTCCTGCAG
GACAACCAGAGGATGCATCACGGGAACATGGATGATTTCATCAAATCCTTC

EsigGOBP13

GATGACAATGGTGTGTTCACTCCCGCACTGAGTGAGAAAGCTGGACGCGAGCTGTATGCT
GGTGCTCCCGAAGAAAAATTAACTAAGCTCAAAGAAGTGAGCGAGACCTGCACGAAAGTA
AACGACTTAACCGTAAGTGACGGTGCGAAGGGTTGTGATAGAGCAGGACACATCTTCAAA
TGCCTCCACGAAGAAGCTAAGAAGGTGGGC

EsigGOBP14

AAAGCTTTGGATGAATGTCGTGAGGAGAGTAATCTAACACCGGAAATAATGGAAGAGTTC
CTCCACTTCTGGAGGGACGACTTTGAGATCAAGCACAGGGAGCTGGGTTGCGCCATCCTC
TGCATGAACAAGAAGTTCGATCTCCTGCAGGACAACCAGAGGATGCATCACGGGAACATG
GATGATTTCATCAAATCCTTCCACCAGGGCGAGAAGTTGGCCCGTCGCATCATC

EsigGOBP15

GAAATAATGGAAGAGTTCCTCCACTTCTGGAGGGACGACTTTGAGATCAAGCACCGGGAG
ATGGGTTGCGCCATCCTCTGCATGAACAAGAAGTTCGATCTCCTGCAGGACAACCAGAGG
ATGCATCACGGGAACATGGATGATTTCATCAAATCCTTCCACCAGGGCGAGAAGTTGGCC

CGTCGCATCATCGAAATC

EsigPBP1

ATGAGCATTGAGGGTTCGAAGTTCAACGGGGCACCTGTGACAAATTCGACGACATCCGAT
CCAGAAAGATCACTTATTGGAGATGATGACAACCCTAATGAGAATTCGGAGGCTTCTAGG
GAGCTGGATTTGGACGAAGTAATGCGGGATTGCAACAATTCGTTTCCTATTAAAATGGAA
TACCTGACATCTCTTAACGAAAGTGGTAGCTTCCCAGACGAAACTGACAAGACGCCAAAA
TGTTACATACGCTGTTATCTACAACGAACAGGAATTATGGACGCTGGTGGTCATCTGGAC
GCAGAACGGGCCTCTGAGCTGCTGGCAGGTTCCCGACCTGGCCGTGAAATCACAGATCTG
CCTGAGACAACAAGCGCGTGCACCGATCGAGATGAGAAATGCCTATGCGAGAGGGCGTAC
GCCTTCATCCGTTGCGTCATGGAAGCTGAGATCGACCGGTACCACAATGGGCAATGA

EsigPBP2

ATGGCTTTCTTCAAGCTTTTCGCGTTTGCTGTTATTGTCGCTGCTATTAACGCCGCTCCCAGTGA
CCCGACCGATCAGAAACAGACCTTACAGTCGATTGGCAAAGCATGTCGCGTGAAAACTCCGGTA
ACTGACGAAGAATATCAAGAACTCTTGGATAAAAAGCTTCCTACCACCAAGGACGCTAAATGTC
TTTTGGCCTGCATCTACAAGACTGTAGGCATTCTGGGCGATGATGGGCTCATTTCCGAGGAAGG
TTCCCTAGCCTGCGCAGAAAAGAAGTATAACGACGACCCAGTCAAAAAGGAGATCCTGTTGGAA
GTTGTCAAGAATTGCCATAAAGTGAACTCTGTGGCTGTGTCCGACGGAACCGACGGCTGTGAA
CGAGCCACACTGAATCTTGAATGTATGCTCAACGAGAACAGCGCGATTGTGACCGGATTCGACT
AA

EsigPBP3

ATGCAGTGCAAATATGGTTTTAGAGAGCTTTTTTCGCTTATTTTTCTGGCGAATATTGCT
CTAATTTCGAGCAAGCCGGATAAGGAAGTAATGAAGGATGTAACTATTGGCTTCGGCGAA
GTGTTGGAAGAGTGCAGGGAAGAGAGCGGCCTGAAGGAGGACGTGATGGAGGAGTTCGTC
CACTTCTGGCGCGACAGTATCCAACACCGGGAGCTCGGCTGCGCTATCTTCTGCATGGGC
AAGCGGCTGAAGGTGCTCGACGATCGTGGTCGCATGCACCACGAGAACGCGGACGCCTTC
GTGAAGTCGTATCCTAGAGGTGAAGTGATAGCCAAACAAGTGATATCCATAGCTGACAAC
TGCGAGAAGAAGTTCGATAGTAACGATGACGACTGCGCGAGGATGCTGATGATAGCGCAG
TGCTTCAAGGACGATTGTATCGAGGCTGGACTGGCTCCCACGGTGGACATGCTGGTCGAA
GTCATCCTTTAA

EsigOR1

TTTCAGCCGCAGCAAGTGGTGAAGAATGCAGCCAGGAATCTAACGGTCGTAACGTGGAGT
GTTATATCAGTAGCATTGTCTATGGTCTTCTTTTGTGGCACAGCCCCATTGATATTTGAT
TCTGATCTCAGACTGGATGCCGACAACGTGACGTGGATCACAAGTCGCCCGCTGCCTCTC
AACACTTGGGTCCCTCTCGACGCAAGAAATCTCACGACATACAAATTCATTTACGCTTAC
CAGCTGGTGTGCCTTACCGAAACTGCCGTGATGATATTGGGCCTGGACACAATATTTTTC
GGTCTCTTAGCTCATGCTACTGCCCAATACAGAATACTGAACACGGCGATAAGAACAGTT
AAGGAACGGTCTGTACACCTTGTAACTCAACGCTCAAAAGGCCTGGCTTCAGAAGATTCT
GGTAACACCATCGATAGAACTGTCGAAGAGGAAATGAAACGACAATTGAGGAAGTGCGCT
GTTCACCATCAAATTATATTAGGATTCTGCGACCGCATAGAGCAAACGTTTCAAGGCGTG
ATGCTCGGTCAATTCGCCGCCCCACTGATTATTATTTGCGTGACCTTGTACCAGTGCGCG

**ACAAGATCCGGTATGAATCTTGACTTCGTGTATATGATTCAGTACCTGTTAACAATTACA
TTGGAACTCTTCTTCTACTGCTGGTTTGGAAACGAGCTAACTATAGAGAGTCAACGCCTA
TCGCAAGCGGTGTTCGACAGCGAGTGGATGGGGGCACCACGGGAATACACCTCTATTTG
AGGATCATTCACGCGAAGAGTTTGAAGCAGACCACACTTACAGCA**

EsigOR4

**ATGGCCGTAGTATATCCGAGCAGCAGTTTGGATTTACTTTTTAGGATTTTGACCTTTTGT
GGGGTTTGGATGCCGCAGTCTTACGTGGGAACGTGGAGAGGGTCCTTATACAAATTCTAT
TCATGTTTTATAATGATGGGAACGATTTTTGCTAACCCGTTTACTCAAATTATGGACCTA
TACATGAATTTCGGCGATTTAGACAAAAATGTGGAGACATGTAGTGTTGCCTCATGCGTT
ATAGCAGCGTCAGTTAAGACAATCAACATAATTATAAGGCGGAAGCAGATTCAACAAATC
TTGACAATGTTGGACAGTGACATTTTCACCCACGTTAGCGCGGAAAATAACATTTTAATG
AATAAGTACATGAGAGCTGTCGAAATCGTCTCAAAGTTGTGCTTATACTTTGGATTGTCG
AGCATTATCCTGTGGGGGGCTCTGCCACTATTCAACAAGTTGGAGGACAGAAACTTACCC
TTGAGGGCGTGGTTTCCTTGGGACACATCTGAATTGTCTACTTACATTATGCTGTATACA
TTTCAAGTGTTCAGCGTCGCCATCTTGGCGTCTTTCAATGCTAGTCAAGACGTGTTGATG
GGTTCAGTTGTTATTTTGCTTTGCGGTGAACTGGATATGCTATGTGCTGAACTTTCGTCT
TTAGGTCAAGGTCCTCGGGTCGCAGAAGAAACGTATGAAATCATCCCCTATTCACCGATC
GGAATTATCGCTGAAAGAAGAAGGCGTCGAGATGTAAACATGCGACTAAGTCGTTGTATT
TTTCATCATCAAGAGATCTTGAAATGCGTCAAACAAGTTGAGAGCGTGTTCAACGGAGCC
ATTTTCATGCAATTTTTACTAAGTTGTATGACAGTCTGCATCGTAGCTTTCCAACTGAGT
GTGACCAAATTCTCCGTGCAGTTCTTGTCGCAGATCATCTACATATGTACGCTTATGATG
GAACTTGTAGTATTCTGTTATCCTGGTAATGAGATGATGGCAAAGAGTCAATTGATATCG
TCTGCTATTTACCAAAGCCCTTGGTACGACCAGGACATGGAGTTTAAGAAGATGATGTTC
ATTCTTATGGAGAGATCTAAACGTCCCGTCATACTCACCGCTGGACACATTCTGCCCCTA
ACGTTGGAAACATTAATTTCGATTCTCCAGTCGTCTTATTCTTATTATCTTGTGCTGCAA
TACTTGTATTCGGAAGGTTAA**

EsigOR6

**TTTTTTGACTACATGCTGGGCCGGAAAGTTAAGATGCTCTTCCCCTACATACTCTGGTAT
CCCTTCGATCCTCGTACATACATCAGATACCCCTTCGTCTACTTGGTGCATGCGGGTGCT
GGTTTCAGAGCTGCTTGCGGAATTGTGGGGCCAGATTGCATACTGGTTACATTGACAAGT
CATATGTGCATGTTATTCGCCATACTACACAACGACTTGGAGAATTTGATGGTGGAATGT
GACGAAGCAACATTTGACAAAACGCTTGTTCAATACAATCAAAGCAGTGTGGCTCAGCTC
AATGAACATGTCAATTCTTCTCTTTATGAACGCCTTGGTGCTTTTGTTAAGAAACATCAG
ATTTTGATAAGTGCTGCTGAAGATATCGAGCAAGTATTCAACCTGAGCATTCTCATTAAT
TTTCTTGCAAGTTCTGTCATAATATGCCTCGTTGGATTCGAAGCAACCGCCAAAGACATG
AAGGAGCTGGTGAAGTTCATTATGTTTCTGCTTTCGTCACTCGTCCAGCTTTTCATGATC
TGTTGGTTTGCTGACAAACTTATGGAAACTAGTGCAGGTTTGTCGCAGGCCGTCTACAAC
AGCCGGTGGTATGACATGCCGATGATCATTAAAAAGGCGTTATTTTTCATAATGGTCAGG
TCTAAGAAACCATCATACATCACTGCCTTCAAGTTCTCCTCAGTGTCATTGAACAGCTTC
ACAAGGATTCTTAGCTCATCCTGGTCATACTTCACTCTCCTGAGGACTATGTACGACCCA
TCCCACTCAACCTTGAAACACTAA**

EsigOR7

TTTTTTGACTACATGCTGGGCCGGAAAGTTAAGATGCTCTTCCCCTACATACTCTGGTAT
CCCTTCGATCCTCGTACATACATCAGATACCCCTTCGTCTACTTGGTGCATGCGGGTGCT
GGTTTCAGAGCTGCTTGCGGAATTGTGGGGCCAGATTGCATACTGGTTACATTGACAAGT
CATATGTGCATGTTATTCGCCATACTACACAACGACTTGGAGAATTTGATGGTGGAATGT
GACGAAGCAACATTTGACAAAACGCTTGTTCAATACAATCAAAGCAGTGTGGCTCAGCTC
AATGAACATGTCAATTCTTCTCTTTATGAACGCCTTGGTGCTTTTGTTAAGAAACATCAG
ATTTTGATAAGTGCTGCTGAAGATATCGAGCAAGTATTCAACCTGAGCATTCTCATTAAT
TTTCTTGCAAGTTCTGTCATAATATGCCTCGTTGGATTCGAAGCAACCGCCAAAGACATG
AAGGAGCTGGTGAAGTTCATTATGTTTCTGCTTTCGTCACTCGTCCAGCTTTTCATGATC
TGTTGGTTTGCTGACAAACTTATGGAAACTAGTGCAGGTTTGTCGCAGGCCGTCTACAAC
AGCCGGTGGTATGACATGCCGATGATCATTAAAAAGGCGTTATTTTTCATAATGGTCAGG
TCTAAGAAACCATCATACATCACTGCCTTCAAGTTCTCCTCAGTGTCATTGAACAGCTTC
ACAAGGATTCTTAGCTCATCCTGGTCATACTTCACTCTCCTGAGGACTATGTACGACCCA
TCCCACTCAACCTTGAAACACTAA

EsigOR8

ATGCTGATTTTGAGGAACTACGTTCTAATAACTGGGGCGTGTACTCTGGCGTATCTGGGG
CCAACTGCATTCGTTGAAAGCGTTGAAGTCGAGGAAGGTGAGACGGATTATTGGGGAATA
GTAAATCTGTACGCGCCTTATGATTATATTTTTTTCGAAAGATATGGCATTGCCAGAATA
GCGATAACGATGTTACTCTACTACCATTCGTATTGCCAGATATTTCTGGATATGTTCTCA
GCATCCCTGTTTGTGCATCTCGGTAGGCAGTTCGAGGCTGTGGCTGACATGTTCTTTAAT
GTACAGTATTCAGATAACGGCGAGGAGTACATGGTTCCTTTGAGGGAAGCTATTGAATAT
CACCAGCTCCTGTTAAGATACTCGACTGACTTGCAGCGCATGTACAATGCAGCGTTTCTG
GCGCAACTCCTGGGATGCTGCCTGCCGCTTTGCTTCTGCATGCTGCTGGTAGTGAACAAC
ATGAAGTCAGAAGAAGGGAATTTGATGAATGCCATCACTCATTTAACGTTTCTGATCGTA
GTGGTGTATCAGCTGTTTATGTACACGAGGTCGGCGGACGTGATAAAGGAAAGGGCTCTT
TTAGTATCGCAGGCAGTATACCTGAGCGATTGGCACCTGGCTCCTCCTTCCACGAGGCGA
GATCTGATCTGGGTCGTGGCGCGAGCTCAGCGTCCCATTGAATACTCGGCGCTGGGAATA
TTTCCCATGACTATTTCAACGTTCACTCAGATAATGCAGAGTACTTATGGCGCCTTCTCC
TTGCTGACCAACGTAGACTGA

EsigOR9

CAAGGCGTCCAGGACACACTGCTATCCATCGACATGCTGACAGCCGATCGTCCCACGCAG
AAAGAGGTGGATCATTTCATCCAGGCAATCGAGATGAACCCCGCCATCGTGAGCCTGAAA
GGCTACGCCCACGTCAACAGGGAGCTCTTGACATCGGCCATGAGTACGATAGCCATCTAC
CTGATCGTGCTATTACAGTTCAAGATCTCCTTGCCGAAGGAAGCC

EsigOR10

TTCTGGACAACCGTATTCGTCTACATTGGCAGTAGCTTCATTCTGTGCTCTTACAGCTTG
TTACTCCACACAGCATCCAGGGATTTATCAGAAATGACTCGGCAGGTTGCTTTGGTCATA
CCTTTGACTGTTATGTTGGGAAAGATGTGGTTGTCAGTGACAAAGAAGGCCGAATTCCAG
TCAATTATAGAAGAAATAAATGACGATTACATTGAATTCGTTCATGCACCTAAGAAATAC
AAACGAATCTACAGAACCGTCCTCGATGAATCACTAAAAGCCCAGACCGCCTGGATACTC

ATATCCAGCATCGTGGCCCTAACCTTTCCAGCGGTAGCGGCCTACAAGATGCTCAACCAC
AATCTGCACCACAACGATCACCAGAAATATATGCTGTACGAGCTGTCGATACCGTTCCTT
GAACCGTTCAAGTACAACACGCCATTTTTCGAAATCATCTTCGCTTACTCCTTTCTGATC
GTCTGCTTTTGCATAACCAATTTCACGGGTTTCGACACCACGTACCTGATTGCGGCTCAA
CACGGAAGAGGTCAGTTGAAGGTCATGACGACGAGGCTCGCTGATCTGTTTAAGATGAGT
TTGGACTCCGACGCCCGCTACACGCGCTTAGGGATTATCGTACAGCATCATCAGAAGATA
TATAGGTACGCCAAGAGGGTCCAGCGTTTCTACTCCGCCTGGTTCACCACTATAGTTTGC
CTCTCCTCGGTTCAGATTTGCTTGACGCTCTACCAAATGACTGAGGCTCTCGCCAAGCAC
AAGGTCAAGATGGAATTCATTATCTTCACAATCGGTACGGTAATGCAAATCTACATGCCC
TGCAGGTACAGTGCTATCTTAAGTGATGCGTCATCTGAAGCGGCTGTTGACATTTATCGA
TGTGGATGGGAAAATAATTTGAATAAAAAAATATGTTTTGCTCTCAAATTCATGATTGCG
AGAGCTCAAATGCCCATTAAGTTTAATGCTTCGTTCATTTGTCCTATGGATATGAATGGT
TTCACATCAATCATTAATACCTCATATTCATACTTTACTCTCTTGCGAACGAAGAAATAG

EsigOR11
GTCGAGGCCACTTATCACATAAATTATTTGGTCCAGATATCAACCAGCGTTGGAGTCATC
AGCATCACCTTGCTGCTTCTCGATATTGTAGACACACAGTCCGTGCAGTTCTTTTCGATG
GGATCGTACATGTTCGCCATGCTTATTCAGGTGTACGCTGTCACGTGGTGTGGGAATCAA
GTTACGGACAAGAGTTCCGAACTAAAATACGCCTTCTACGACAGTCCGTGGTATGTAATG
AGCAAGAAATACAAGCAACTCACAATGATCGTGCTGCAGAAGCTGCAGACGCCCATAATC
CTGAGAGCCGGCAATTATGTTCCACTTAGCCTCATAACATTGGTTTCTATACTCCGGACT
TCTTACTCCTACTATGCAGTACTGCAGCAAAGCAAGCATAAGAAAACTGGTCTGTGA

EsigOR12
GTTTATCAGCTCTACCAGCTCTACCTGATAACTTCGTGTGCAGATGAGGCGCGCCATGAT
GCGTTGCTCGTGGCGGAAGGCGCATACTTCGGTGACTGGACGAGCGGCTCTGTGGCAGCT
CGGAAGGCGCTGCTGAACGTCATACACCGAGCGAACAAGACCATCTCTTTTTCTGCCCTT
GGCTTTTACGATATGAACATGGAGACTTTTCTTGCTATAATTAAAGCTGCTTATGGAGTG
TTTGCA

EsigOR13
ATGATGACCAAATTCAAAGTGCAGGGCCTGGTGGCCGACCTGATGCCCAACGTGACGCTC
ATGAAGGGAGCGGGGCATTTTCTTTTCAACTATTACGCCGAGAATGGGGCGTTGTCCGTA
CTCCTGCGCAAGGCATACAGCGTGATGCATCTGGTGCTGGTGGTGACCAACTTCCTCTGC
ATTGCAGCCAACATGGCCATGCACTCGGACGACGTCAACGAGCTCACTGCAAACACTATC
ACACTGCTCTTCTTTACTCACTCCATCACTAAGTTCATATATTTCGCGCTCAATTCCAAA
TCCTTTTACAAAACATGGGGAATTTGGAACCAGGCAAACACGCATCCCCTTTTCGCCGAG
TCGGATGCTCGCTACCACCAGGCAGCCCTCACCAAGATGCGAAGGCTGCTTTACCTCGTG
AGCGCAGTCACTATATTGTCAGTTATAGCGTGGACAACAATAACATTCTTTGGTGAGTCT
GTGCGGTTTGCGCTGGACCGTGAGACAAATGAGACAATTACAGAACCCGTCCCACGACTC
ATGGTCAAAGCCTGGTACCCCTTTGACGCCATGCACGGCCCAATGTACATCATTGCGTTT
GTCTACCAGTTATACTGGCTGACCTTCACGCTAGCCCAAGCCAATTTGCTGGATGTACTC
TTCTGCTCGTGGCTCATCTTTGCCTGCGAACAGCTCCAGCACCTGAAGGCCATAATGAAA
CCTCTAATGGAGCTGAGTGCCAGCCTCGACACATACAGGCCGGGCACTGCTGAGCTGTTT

CGCGCTCATTCTGCCTCCTCGCAATCAGCTTTAATTAATGAAAAGGACGAAAAGAGCCCC
GAGGGTGGAGATTTCGATATCCGTGGCATCTACGCAACACGTCAGGACTTCGGAATGGCG
GCGCGCGGAGAGCCCACGGCCAATAACCCCAACGGGTTGACGCGCAAGCAGGAACTGTTG
GTTCGCTCAGCCATCAAGTACTGGGTCGAGCGGCATAAGCATGTTGTTAGGCTGGTTGCA
TCTATCGGTGATACATACGGCACAGCTCTACTATTCCACATGCTTACCTCTACCATCACA
CTTACGCTTCTTGCCTATCAAGCTACTAAGATATCTGGCATTGATGTCTATTCTCTGACT
GTCATCGGCTACTTGGTCTATACGCTGGCTCAGGTGTTCCACTTTTGTATATTTGGAAAC
ACACTCATTGAAGAGAGCTCATCTGTTATGGAGGCAGCCTACTCGTGCCACTGGTACGAT
GGATCAGAGGAGGCTAAAACCTTCGTTCAGATTGTATGCCAGCAGTGCCAAAAGGCCATG
TCCATCTCCGGAGCCAAGTTCTTCACTGTCTCACTCGACCTGTTTGCTTCTGTCCTTGGT
GCCGTCGTCACATACTTTATGGTGTTGATTCAACTCAAGTAG

EsigOR14
ATGTGTCTGTTTCTGTATTACGTTTTTATTAGACGCCGCGACTTGCTTAAACACATTTAC
GACACTCTGTTTAATAAGCTGTACATCCATATTCAGGTAGGGGGCAAGGAAAAGGTGTCC
CTATATAAAAAGGCAAGCCAACGTGGGCATTTTTTCATCAATTTTTTCTTCATTCAAATA
TTATTCACTGCAACGCTTGTTTTTGTAAGGCCAATCATCGCAAGTGGAAATCAGTTTTTT
CTCGATCCCAAAATTTCTTCTATTCCTGCTTTGCCACCTGTATCGTCTCCAGTCAGGGAA
ATTACTATTTGTATGCAGGTTATCAGCCTTTTTGTCATCGCTAGCTTAACATATATTGGA
TATATTTTATTCTTCATCTTGCTAATGCATGTTTATGCGCATATCTATATTTTGGGAGAA
GATATACAGAAAATTAAAAACCTAAAAAAACAAGAAGACATTGCGAGTGCTATAAAAGTA
TTTGTTGAAAGACATCAAGATATAATAACGTGTGTTGCTGATATGACCAAGCTCTACGAA
CCCAAGAATTATATATTACCGTCCTTTATTATGCTCATCGTTCTTTGTCCCGCAATAGGG
TTTGATGGGACGACTGACACGATATCTTCTGCTGTCCTGGTATACTCACATGCCCTCCTA
CTGTTCAACTACTGTTATGGTGCTGATATCGTCGTCCAAGAAAATACGGAGCTGCTGAAT
AAAGTAGCTTGGTGCGGTTGGGAAAAGTTTGACCGAAGCAATCAGAAGGCTATTCTTATT
GTGATGTATAGGCTCAATAGACCGCTGAAGCTCCGAGCCGGCATTGCTGGAAGCTTTTCT
TTGGAAATGTTTGGACAAACTATGAAAGCCTGCTATTCTGCTTATGCAACTTTAAATACG
GTCAAATAG

EsigOR15
ATGAAGTGTGCCGAAGGCTTGCAGACCATAATGAACTATGTGTTCCTCATACAACTTATT
GGCAGCTGTGTACCATTGTGCTTTATCATGTTTTTAGCTATCCAGGCGCCCGTAATGGAT
GATGAGAAAATGGTGGATGCAATTGGTCACTTCTCTTTCCTGTTCCTTGTCGCTTATCAG
CTATTCCTCATGGTGAGGTGCGCCGATGTTATAGTCGAACGGGCCTCTTCAGTTGCCGAG
GGTGTCTACATGAGCAACTGGTACCTAGCGCACCCCAACATGAGGCGACAGCTGACCAAC
GTCATACATCAAGCTAATAAACCTATCAGATTCTCAGCACTGGGCGTGTTTCCTGTGACT
ATGGATACTGGTATTAAGATTGTGAAGAGCGCATATGCAGCCCTGACTTTGATCAGAAGA
CTCGATTAA

EsigOR16
ATGCTCAACGGAGTTTCCTCGATTATATTCGGTATCAGGTGTTTGATTCATATCCTTGAT
CTACTTTTCAACAAGAATAATTTTAAATACGTTTGCAAGCTATTCGCTGATGATATATGG
GTGAGGCAGAATGCAGAGGAACGATCGAAATGCGAGAAGAAATTGAAATTAGCACGTTTG

**GTTTGTTACGGTACCATAGCTTCCGATTTTGCCTACATGATTGAACCGACAGTTCAAGTA
TTGTCTTCAACTATTGATTATGCATCCAAGGGATATACATTAAGTGTACATTATCCGATC
GATACGCATCAACCAGTCGTCCACGAAATAACTTACGTACTAATCAACATTGCTGGATTT
GTCAGGGCGTCACTGATTGCTAACATCAATTTGATGATGAATTATTTTATAGTCTACACT
TCATACAGGTTTGAAGAAATTTCAAAGATACTGATTGTGAAAGATTTAGAAGTTAGTACT
GACAACAAACTGCAACACAAAGAAAAAATTCGCAAAGCAGTTGAAACGCAACAATTATTG
ATAAGTGCCACAAATAAGCTGCAAGAAATGTTCTCCTTAAGTATCTTTGCACAGTTCCTG
TCGGATTCTCTCTTGTTTTGTGTTTTAGGAGCGTTCGTATTATTGTCGGAGACATCGATG
GTGGTTTATTTCAAAGTCTTGCTTTTCTTACTATCAGTGCTCGCGCAGGAATATGTCAAC
TGTGTGGCCGGGGAGTCGCTGACACGATCGAGCGCCGAGGTAGGTGATGTTGTCTACATG
AGCAACTGGCAGGACCACGATCCGACGAACCCGTCAATCGGACTGATCATTCAACGGTCC
CAGCGAGTATTGAAACTGACCGGACTCGGTTTCTTCGATCTTTCTCTGACTACATTTACG
AAGGTATGCAACAGATCTTTGTCGTACCTGACAATTCTACGCACATTCTTCGAGTAG**

EsigOR17

**ATGAAATACTTTGCGTTGGGAGACTTTACGCCGTCAAAAGATATAACGGTTCCCGCCACC
AATAGATATTTGACTTACATTTACATTCTTATCTGCTGCTTGGTTGTTGCCAGCTTGACG
GGGGGAGCCGTGCGCATCGATACATTCAGCGAATTTGTTTTGGGCACCTCCGAACTCTGC
AACCAGGTTCTTGCTGTCGTCAAGTACATTGCCCTCGTTCTCAACCGGAAGAAGGTGTAC
AACATTATTAAGCTGATTGCAATCGACAGGTTAGATTACAGCACTGATGAGACTTTTCTA
GATCGAGCCGCGGCAAAATCCCGTAAGGCATTTTGGGCATACATGGTCATGTGTCTTATT
TGGCTGCTGGGTTACATTGCTGTATCTTTTGTTGGCCAACACAAATCTGTCGTGCGCGAA
ACTGAATGGTACTTTATGCCCATGTACGTGCCATACGATATATTTATTAAAGAAAGATAC
GGTTGTGGCACAATTTTTTTGACAGTGGGATTGATTTACAACGGTTATTGTCATGTTCTT
ATGGATCTTTTCACAGCAACTGTTTTTATGTACACTTCTGCCCAGTTTAAAACTATTGCG
TTGATGTTCGAGAACGTTAAATTCATAAACACAGCTAATGGAAAGTTTGCCTTGAAGAAG
AATAAACATAATAACAACGGCCACAATAAGAACGTGGTGTCAACAATGCCTGCCGATTTT
AGAAATGCTATACAGTACCATCAAATAGTACTGAAAAGCGCTAATGATCTGCGTAATGTT
TACAGCGCCGTATTCTTCTTCCAGATCTTGTCGAGCTGCATACCGCTTTGCTTCAGCATG
TACCTCATAACACATGCGTTAAATTATACTCGTGACAACTTATTCTATTTGCTGATTCAT
GTGAATTTACTCCTGTATTACATGTTCCAACTTCATCTTATCTTGTCGAACGCTGATGAA
ATTAAAGAGGCAGCCTTACGTGTGGCTGATGGAGCATATTTGAGTAACTGGTACGAAGGA
TCATTGCCCACTCGTAGTGCGATGATCAACATCCTCTTGCAATCTAATGAACCCATTGCC
TATTCGGCACTAGGAATATTTGATATCAATATGCAGACATTCATAACGATAGTTCGATCT
GCATATGGGGCTTTAGCACTTATGACAAATTTTACATAA**

EsigOR18

**GACGGAAGCAGCGAAACGATGTCTCGGGAAAGTTACCTTAAGATTCACATAGTGCTGTTG
AGACACTACGGCTTTGGCGACTTCACGCCGGCCAAGGGCGCCGACGTACCGAAGTTCGAC
AAGGGCTGGACCGCATTCCACGTCTTGCAAAAGATCATTTGTCTCATCGCTATGTTCGGG
GGCGCGCTGTATGCTGAGACATTGAGGGGCTTCTCCCTTTGCTGTATCGGATTTCTTAAT
TTGCTGCTCGGCTGCATCAAGTTCGTAGTCCTGTTCGTGCACAGGAATAAAGTTTACAAA
CTCGTGAAATTAATCGAGATAGACCGACTGGACTATTTCGACGATGACAGGTTCATGGGG
CCTGGAAAACGTGGAACTCGCAGGGTCCTTATGGGCTACATGATTATAGCTGTCTTCTGG**

TTAATAGGAAATTATGTGAGCTCCTTTAAGAATGACGTGGACAGCAAGTGGACCTTGATG
TACCTGCACATACCCTACGACTTCTATGTGAAAGGCAGGTATGGCATCATAGAGTTCCTT
GTTACGACGCTCATTATGTACCAAGCCTACAGTTTGATCTTCACGGACATCTTCTCTGCC
TCCATCTTCATATACATCTCTCAGCAATTCAAGACGGTGGCTTACATGTTCGAGACGGTG
CGGTACAACGATCCGAGCGAGAGGGACTTGCTGCGCAGAGATACGGAGAACGGGATCGCT
CTGCGCGACGGAAAGGGGCGACCCAAGATGCCAAGCGACCTGAGGACGGCCATTTTATTT
CACAAAATACTGTTAGAGTGTGCCGAGGATCTGCAAACCGTGTGGAAATTAATATTTTTC
TTCCAAGTGTTGGAGAGTTGCTTGCCGTTGTGTTTGTGTATGTACCTCGTTCTGCATACG
GTCAACTTCACTTACGACGAGATATTAGCAACCTTCAGCCACTTCAACTTCTTGATTTAT
CTCCTCTACCAGCTCTACGTGTTGACTGCATGTGCGGATGAGACGCGCCATCAAGCGTTG
CTCGTAGCGGAAGGCGCATACTTCGGTGACTGGACGAGCGGTTCTGTAGCAGCCCGGAAG
GCGCTGCTGAACGTCATTCATCGAGCGAACAAGCCCCTATTATTTTCTGCACTTGGCTTT
TACGATATGAATATGGAGACTTTTATTGCGATAATAAAAGGTGCCTATGGAGCATTTGCA
TTGATGAAAAGTGTCATTTAA

EsigOR19

TCGATGCTGGTGGAGAGCTTCTGCTACTTCATCGTGACGACAGTGGTCATTATCTTGGTG
CCCAGCACGCAACGCGGTAAGGAGAAACTGTATTGGATCATGAAGGTACTCGAGAGAGAT
TTCAAGTTCGCCTGTTCGCGCGTGGGAAGCCAATACAGAGAGTTGTTCTACAAGAAGCAG
CTACTGATATGGCGCATGGCCATTCTGTGGTTCCTCTTCACCTCGCTCATAAACACCTCC
TTCTGCCTAAAGGGTATGCTGGACATCCTCTGCCACGAGCTGCTATCCGACTACGACGAA
TCCTACCAGAGGCCCCTCATATTCTCCCTCTACCTACCCTACGACGACCCTTACAGGACA
CCCAAC

EsigOR20

GCCGCCATTCCTTACTTTGGATACATTGGAACGCCTTTATATCAAATCACATGGGCTTTA
CAGGCTTTCACCATGACGGTTACGGACGTGCACACGTACTTTCTCGACGCTCTGTTCCTC
GTCACTCTGATGCACATCTACGCCCACATGCACATGCTGGGCGAGGACATTCAGAGCATG
AAGAATATGACCAGACGGGAGGATGTGACGCGCATGATCCGACAGTTCGTGGAGCGATAT
GACGACCTGCAAATCTGCTTAAACAAGATCACGGCCATATACAGCCCGAAGAACTACCTT
CTTCCGTCGTACATCACTGTCATTATCCTGGCGTGCGCCATCGCCGCCAATGGTTCAAGG
GAGACGACTATGTTCGTCATAATGGGCTACGCGCATGCGCTTATTCTATTCAATTACTGT
TACGCTGCTGAAGTCGTCGTGGCTGAGAGCGCTGATCTTCTCCACAAGGTGGCGTGGTGT
GGATGGGAGCAGTTCGACCAACCCAACAAGAATAACGTGCTGATACTCCTCAACCGACTC
CAGAAGCCCGTCATGCTGAGGGCCGGCTTCTCTGGCGGCTTCACCTTGGAGGTCTTCGGG
AATACTATGAAGACCTGCTATTCAGTGTACGCAACTTTGAATTCCGTCAAATAA

EsigOR21

ACAAAATCTGGAAGAAAATTTGACAATGATGCGTCGACGCCCTCTCCAAATATTGAACAT
TTAGATGAAAAGTTAAGACACATCATTATAAAACATTTGGAGATCTTGAAGTTGGTCGAC
AAAATGCGAAATACTTACGATATTGCCATGGGATTAGACTTTGCTTTAATATCAGTCGCC
GTCTGCACCCTTTTGATCAAGATGGATCAATCGGAAATGTTGTTTTTTATCCAATACACG
TCTTACGTATTTGGCGAGGTCATGATGTACTGCTATTGCGGTCAACACTTGATCGATGCA
AGTAACCAGTTGGAAAAAGCGGCGTACGCCTGCGGCTGGGAGGAATGG

EsigOR22

ATGAAAGATAATGAAACAGCATCAAAGTACAAATATTATTCTCACTTTCTGTATTTATGC
AAAGTTTCGTCTTTGCTTCTGAGCTTCTTCAACGTAGCATACAATATCAAAATTAGAAAT
ATGAACGAGCTGATACGGCAATTTTCTATGTTGGTGCTCATCATAGCAGCGCTGTTCAGG
ATTATAATGAGTTCAAAGTTCAGTAAGGAATTACGCGACTTGAAATTGTCGATGAATGCC
GATTACGAAGAGTGGGGGCATTTTGACGATACCCAGTGCGTAATTATGAATGAGCTTATA
AGAGGTAATAAGCGAATTGCTTTAAAGGTAACCCTGATCGGCACGAACGCGGTGATGTCA
TTTATCGTCAGTTGGGCAGGCAAGATGCTGTATGGCGCTATTACAAGCAATTACAAAAGA
CTATTAATATTCGACTTCAAAATCCCGATGTTAGATGAATATCATAAATATCAGCCACCT
CTTTACCAAAGTATCACGATGTACACTTGCCTGATAATGTTCCTGAACATGCTGACTCTG
ATCGACTTTGACAGCACGATGCTGATAACCGTAAATCACTATTGCTACTCGGTGGACGTT
TTGAACCATAGACTAGTTAATTTGTTCCGGGGTCAGGATCCTCGGTCGTACGATCCTCTC
TTGACTGGACGAAGGCTCGTGAATATAGCGCAGCTTCATCGTCACGTTGTAAGCCAATTT
CAAGTGATACAAAACATTTACTATCCGTATTTGTACCTGATTTTCACACTGTCATCCGCC
GAAATTTGCGTACTCATGTTCCAATTCACCGAGGAACTGTTTGCCGGTCGCTTCCAGTAC
GAATTTCTTGCGCTGTTAACTTGTTCAACACTCTTAGTGCACACCATGTCGTGGTATGGA
GACAAAGTTATGAAACAGTGCGAGTCGGTGGGCTCTGCTGCGTATCACAGCGGCTGGGAG
GGTTGGCCGGACATGGCTGCGCGGCGTGACCTGCTCCTTATTATGATGCGTACCGCCAGA
CCTACCACTCTCAGGACCATTTTCAGCGACATCAATCTCGCCGCTTTTAGCGCTACTGTC
AACTCCGCATGGTCCTACTTCACACTTCTACGAACCAGCAGCGAATAA

EsigIR25a-2

ACCGGTATGGGCTCGGAGACCGTCAAGTCTTTCACGGCCGCCCTGGCGCTGCCGACCATC
TCTGGCTCGTTCGGGCAGACAGGCGACCTGCGGCAGTGGCGAGCGCTCGACGCCAATCAG
ACAAAGTTCCTGCTGCAGGTGATGCCGCCGGCGGATATGCTACCGGAATGCATAAGGTCC
ATCATCACCAAGCAGGATATCACGAATGCTGCCATTATATTCGATGACAATTTCGTACTA
GATCACAAATACAAGTCGCTCCTCCAAAACATTCCGACACGGCACGTGATAACACCCCTC
AAAGGCAGCAATCTCGAGGATGTGAAGAATCAGCTGCGCAGCCTGAGGGAGCTGGACATC
GTCAACTTCTTCATACTCGGCGACCTGAAGACGATCCAGCGCGTGCTCGACGCCGCCAAC
GAGAACCAATACTTTGGGCGGAAAACCTCTTGGTACGCGCTGAGTTTGGAGAAGGGCGAT
GTGACC

EsigIR13

CGTAAAGATTACCAATTCGTTTCTAATTTGTTTCCACAAAAATTGAACAATTTTAATAGT
TGTCCATTTATTATATCAGCTCGTGTTACACCATCATTTTTTACTGTGAGAACTGACAGT
CATGGAAAACAAGTAGTTGGTGGAATAGAAGGTGACTTTTATAACGTTCTGTGTAGGATG
ATAAATGCAACGTTAATAATATTGGATGCAAGTAGACCTGCTTCATCCAATGAAATAAAT
GGA

EsigIR18

ATGAAAAACGATTCAATGTTCACTAACTACGGTAAATTTATAATCATTTGCGATGAAGGT
GATCAAACTGCGGAACGCTGTAGCGAGGAAATCTTATTTGATACTTGTTGGAGGCACTAC
ATAGTAAACGTCGTACTTTTACATCACTCTGAAGCAGACGAAGTGATTGTATCTACATAT

TTTCCCATGGAAGATGGAATCTGTAACACAGTAAAGCCGAAAACATTAGACAGCGTAAAA
CTACGTAAAGATTACCAATTCGTTTATAATTTGTTTCCACAAAAATTGAACAATTTTAAT
AGTTATCCATTTATTATATCAGCTCGTGTTACACCATCATTTTGAACTGTGAGAACTGAC
AGTCATGGAAAACAATTAACTGTGAGAATAGAAGGTGACTTTTATAACGTCCTGTGTAGG
ATGATAAATGCAACGTTAATAATATTGGATGCAAACAGACTTGCTTCATCCAATGAAATA
AATGGAGTGAGACTGATGGATGACCTCATGCTACGTCGAGCCAATATGTCCTTTGCCAAA
GTTATTCACGTTGAAATTGCGAATATTTTCCGTTTCACATATCCTGTCTGGGTCACGAAG
GTCGGCTGGGTCTCTCCACTTATTCCCCTTCGGCCTGCGTGGGAGAAATTAATCACGCCG
TTCGACTTGCAGCTTAGACTTTCATTCGCAATCGTTATCTTGGCATTGTTTCTCGTATTG
GTGGTCTTGAAAGAAACGAGGTTAAAACGTAAATGGAATGGGTTCTTTAAAACAGACGTA
TTGAATTCCAACCTCTATATCAATACTTGTGCCACATTCCTTGGTCAAATAACCGCTAAA
CTGCCAAGGAAAACTGCAGTTGCGTGTTGTTTAACCTTGTGGATATGGTTCAGTTTCTTC
ATGCTGAATGCTTACCAGGGAGCTCTCATCAGTGTTCTCAAACGTGACTCATACCAATTG
GATCTGGACACTGTTTCTGGTGCCATTAAGGCCGGGTATGAATTTAGATGAATATCTGAG
TTAAGGAATTATTTTTTTGAAGAACCTAAGTTGTATGAACAGTACACTGTTATGGATATT
CGTAATATCATAAAATCTTTGGAGTTAATGTCTAGAGAATCTTTAAAACTAATCATACAT
ATACCTTTAGATTTAATAGGCTACTTAAATTTTAGCAAACATCACATTCGATACTTGCAT
GTTATTTCAAATGAAAAAAAGCAGCAGCACGTTACGTATTATTTACAGCCAGACTCGGCG
CTGGAGAACACATTAAACACCGTCGTTCTTCGTATTGGAGCCAGTGGAATTTTTGTCAAA
GTCACCGCTGGAGCACGTTATTTGTATAATATAAATAGTTACAAGATTTATTGGAATACA
GAGCGAGATAATGCATTGACTATGCACCATCTGGAAGCGTGCTTCTTGCTTATTATTTGT
GGATGGATTTTGTCTTCGTTACTTCTATTCGTAGAAGTAATTTATAAACGACATGAACAA
TATCGAAAATCAAAGCGCCGTTCATTGAGAAGATGGATGATATAA

EsigIR75p-8

ATGCAAAAAGAAGGGTGTTATAAGTTTAACCTGTGTATCTGTGTACTTGTCTGTGGCATC
TTAGCTCCCAAACGGATGGACCGATTTTAATTTAGTTTTTTTGGTTGAAAGGTGACTTG
ATCGAGAGTGTTCTTAGCTATGATTCAAGAAAATCTGTTTAGCCTTTAAAGATCATCAGC
TCTTTTCTGGTTGATGAAAGAATGTGA

EsigIR19

GGTGATTTGTACTTGCCTCTGTCCAAAATGATCAATGCGACATTGCAAGTGAGGGTGGAA
ACTGTTCCATGCTTGTTTAATGGAACACACTGGACTGGAATACTTGAAAATCTCGTAATG
GGACAAGCCAATTTTACAATTGCTAAAATTTTAGATATAGACGTAATAAACACTTTCCGC
ATAGTGTACCCTTACTGGATTAAGGAGATAGGGTGGGTCTCCCCTCCTGTTCCTCTCAGG
CCCGCGTGGGAGAAATTAATAAAGCCATTCGACTTCATACTCAGAGTTTCATTTGCAATC
GTCGCATTGCTCTTGGCTCTTGTATTAGTGATTTTGAAAGAAACAAGATTAGAAGGTAAA
TGGAATACATACTTTACAATGGACGCCTTACATGGCAACCTCTATATAAAGACCTGTGCG
ATATTCTTTGGACAAGTAGCTGCTAAGCGACCAATGAATGTTTCACTGTCATGTTGTTTA
GCTCTGTGGATGTGGTTCAGTTTCTTCATGCTCAATGCTTACCAAGGAGCGCTCATAAGT
ATTCTGAAACGAGATGTGTACCAATCTGATTTGAACACTGTTTTGGGCGCTATTGAGGCT
GGGTATGAATTTGGAGGACCGACTGTCTTGAGATCATATTTTACTGAGAAACCTAACCTT
TACGATCAATACAAGCTTATAGACTTTCCGATATTGGATAAGTATTTGGAATTAATATCT
GAGGGGTCCTTAAAATGTATTGTACGTGTATCTCTGGATTATGTGTCTCACTTCAATTCA

ATAAAAAATCGCACTAATATCTTGCACGTAATTCCACATGAAAGACAGCAGCGGTACATA
GCAAGCATTTTGCAACCAGAATCGCCATTAGTAGACACCTTAAGTACCATTTATTCTCGC
CTGGTAGCTGGTGGATTTTTGCAAAAACTTGAAGCTGAATTCTACCACTTAAATATGATG
GAAGATCTTAAGCGTCAATGGAACAGCGAGAAGGACGTCACACTGACTATGCACCACATG
CTGGCGTGCTTCTTTGTTATAATTAGT

EsigIR75a

CTAGATCTCATCCAACAACAACATAATTTTACTTTAAAATATAAAGTAGTTGATCGCTGG
GTTGGACCAAGGGATAATACCACTTCCATTCAGAGCCTCGCCCGGACCATGGCTCACAAT
GAGGCAGACGTAAGTTCCACGTGTTTGCGGTTGTTCAGTGAACGTTTGACCTGGTTTGAT
GTACTCATGCCGCATCTCACAAAATTAGAGACGCGACTGTATTATCGCATCCCTGACGTG
GTTATTGGCAATTACGACAATGAGTTTGCACGTCCATTC

EsigIR14

CTGCGCGCCGTGAGCGTGGAGGGCGAGGCGGGCCGGCCGCGCGTGCAGTTCACGGCCGAG
GGCGAGCTGCGCGCGGCCGACCTGCGCGTCGTCAACCTGCGGCCCGGTGTCGGCGAGCAA
TTGCGCTGGGAGGAAATCGGCGTTTGGCAGTCGTGGCGGCGCGAGCAGCTCGACATCAAG
GACATCGTGTGGCCGGGCGGCAGCCACACGCCGCCGCAGGGCGTACCCGAGAAGTTCCAC
CTCAAGATCACCTTCCTCGAGGAGCCGCCTTACATCCAGCTCGCGCCGCCAGACCCCGTC
AGCGGCCGCTGCCCGCTCGATCGCGGCGTCTTTTGTCGCGTGCCCTCTCATGCGCCGGAC
CTCGGGGTGGAGGCAAATAACAGCTCGCTGCATCAGTGCTGCTCAGGGTTCTGCGTGGAT
CTGCTTGAGAAGCTGGCTGAGGAGCTGGGTTTCACGTACGAGCTGAGTCGCGTGGCGGAC
GGCCGCTGGGGTACCATGCAGCGTGGACGCTGGAATGGCCTCGTCGCCGATCTTGTCAAC
AGAAAGACCGATATGGTATTGACTTCGCTGATGATCAACTCGGAGCGAGAGGCGGCGGTA
GATTTCAGCGTGCCATTTATGGAGACGGGCATAGCGGTGGTGGTGGCGAAGCGCACGGGC
ATAATTTCGCCAACGGCCTTCTTGGAGCCCTTCGACACGGCTTCGTGGATGTTGGTGGGT
GCAGTCGCCATCCAGGCGGCCACCTTCACCATCTTCCTATTCGAGTGGCTCTCGCCCAGC
GGGTTCGACATGCGCGTGCGCGGCGCTGCGCCGCATCGCTTCTCGCTCTGCCGCACGTAC
TGGATCGTGTGGGCCGTGCTCTTCCAGGCGGCCGTCCACGTGGACTCGCCGCGCGGTTTC
ACGGCGCGCTTCATGACTAATATGTGGGCCATGTTCGCCGTCGTCTTCCTCGCCATTTAC
ACGGCCAACTTGGCCGCCTTCATGATCACGCGCGAGGAGTACCACGAGCTGTCGGGCCTA
GACGACGCACGGCTCGCGCGCCCGCTCTCCATCCGCCCACCGCTGCGCTTTGGCACCGTG
CCCTGCTCTCACACCGATGCCACGGTCGCCAAGTACTTTCCTGAGATGCACGCCTACATG
TCTAGTTACAACCGGAGCACAGTGGGCGCTGGCGTAAGCGGCGTGCTGTCAGGAGAGCTG
GACGCGTTCGTGTACGACGGCACGGTGCTGGACTACCTCGGCTCGCAGGACGAGGACTGC
CGGCTGCTGACGGTGGGCGCGTGGTACGCGCGCACCGGCTACGCGCTCGCATTCGCGCGC
AACTCTAAGTACGTGCCCATGTTCAACCGGCGCCTGCTCGAGTTCCGCGAGAACGGTGAC
CTCGAGCGGCTCAGGAGGTACTGGATGACGGGTACGTGCAAGCCAAATAAGCAGCAGCAC
AAGTCGTCGGATCCGCTGGCGCTGGAGCAGTTCTTGTCGGCCTTCCTGCTACTGATGGCG
GGCATCCTGTTGGCGGCGCTGCTGTTACTGCTCGAGCATCTTTACTGTCGTTATGCGCGC
GCGCCGCTCGCCGCTAGTCGCGCGGGATCCTGCTGTGCGCTCGTCTCGCTCAGTATGGGT
CAGTCGCTGACGTTCCGCGGGGCCGTGCTGCGAGCGGCGGCGGCGCGTGCGCGCGGCCGC
TGCCGCTCAGCGGAGTGCGGCGCGATACTGTGGCGCACGCGGCACGAGCGC

EsigIR15

ACGCTGCGCGGAATGTGCCCGGACGCAGATGACAACGAGGGCATTACACTGGAGAGTCTC
GGTGGAGTGTTTATCGCGACTCTGTTCGGGCTCGGCTTAGCCATGATCACACTAGCGTGG
GAGGTCTTCTACTACAAACGCAAAGAGAAGTCCCAGTCCAAAGTGACAGACTTGAAGAAA
GACACCGGCAAGACGAGTAAAATTGCAAAACCCGCGACAACTTTCGATTCCAAAATGCCA
AAGAGGAATAAAATC

EsigIR16

ATGAGCGTGGGCGTGGAGCTTTTACTTTCGACAATATGCATCAATGCCACATTTTGCCCT
GATGTTTTTGAAGAAAATCGTCCATTCGAGAATACGTCGATGGCAGATTTGATTATAATT
CCCGAATCAAAACGTAATTTACACCTATCTTTGTTAAGAAAAGAACTAAATGGGAAACAT
TTACGTATTGGCACTATTGAAAATTTCCCTCTTAGTTATACAGAGATGGTAAACGGCACT
CGGGTGGGCAGGGGCGTGGCTTTCCAATTTGTGGACGTTTTGAAACGGAAACTTAATTTC
ACTTATACTGTGCGGCTTCCCGCACGGAATTGGGAAGGTGGTTATGGGAGGCTGGAAAAG
TCGTTGCTGGGAATGCTTGCCAATAAAACTATAGATCTGGCTGCCGCCTTCCTCCCAATT
CTAACCGAAGCCCGATCGATAGTCCGATATTCTGTGGCGCTGGATGAGGGCATTTGGATG
ATGATGCTAAAACGACCGCGCGAGTCTGCCGCCGGATCCGGTCTGCTAGCACCTTTTGAC
GAGAATGTCTGGTACCTCATCCTAGTAGCGGTGATAGCTTACGGGCCCTGCATCGCTATC
ATCACGCGCCTTCGACACTACGTAACGGACGACGACACCGAGCCGCCCATAAGCATGGCC
CACTCCTTCTGGTTCGTCTACGGCTCCTTCATCAAGCAGGGCACAACGATGGAACCCGAG
TCCAATACAACCCGAATGCTCTTCGCTACGTGGTGGATCTTTATTATACTTTTGTCAGCT
TTCTACACAGCTAATCTGACAGCTTTCCTAACCCTTTCAAAATTCACACTGGATATTGAC
GGCCCGAAAGACCTTTTGCGCAAGAGCTGGATTGCCAGGGAAGGAAGCGCTGTGGAATAT
GTCATCGCCAACCCCAATGAAGACTTATATTTCCTGAACAAAATGGTCACGTCCGGTCGC
GGTCGGTACAGCAGGCTAACGGAGGACAAAAGCTATCTAAAGGAATTGACCCGCAACGTG
ATCTACATTCGCGACAGGCCAAGCGTCAACCACCTAATCTACAACGACTACCGGGAGAAA
GCACGAAGCGGTGTCGAGGAAAAATCGAGATGTACATACGTCATCACCGCCAACCCCTTC
ATGATACGATCTCGGGCTTTCGCTTATCCAATTAACAGTACTTTACCGGCACTGGTCGAT
CCAATACTAAGATCGTTTGTGGAGGGTGGCATCGTTGAATACCTCGAAGAGCTAGATCTT
CCCGCAAATGAGATTTGTCCACTCGATTTGCAATCAAAAGAAAGGCGTCTCCGGAACACC
GACCTCACCATGACGTACACATTGGCTGGCATTGGTCTCTGCGCAGCAATCGCTGTATTT
GTAGGAGAGTTGCTGCATCGATATTGGAAGGCGCGACGCGGTGACTACGATGATGAAATC
GACAATCAGCTGTTTAAAAAGAACAAACAAACCAAGTCTAAAATATTTTCGGAGCGTACT
CGACCTCCGCCGTACGAATCGTTGTTCGGATCTAAAGACAAGAGTTCATTTGAAAACAGC
CAGAAACGCATCATCAACGGCCGCGACTACTGGGTCATAAAGAATTCCCTGGGCGAGGTG
CGACTCGTGCCAGTTCGAAGTCCCTCCGCGTTTCTTTTTCAATAA

EsigIR17

AGTCGAGACATGGTGAGCCGCGTGATCCAGTCGCTGACGTCGCGCGTGGACGACGCGAGC
GTGAGGCCGGTCTCCGTGACCGTCTTCAAGATGCGCCACGAGGACACCGACGCCAAGCGC
CGCGAGGAGATGACGCGCGTCCTCTCCAAGCTGCCACTCAAGCACATAGGCGAGAACTTC
ATCGCCATAGTGACGTCGGACGTGATGGCCGCCATGGCGGCGCAGGCGCGCGCGCTCCAC
ATGACGCACGCGCGCGCGCAATGGCTCTACTTCGTATCGGACACGGACGCACGCGTCGAC
CAACTCGAGCGCTATGTCCAGTCGCTAATAGAGGGCGAGAACGTCGCCTACGTGTACAAC

GTGACGGACGTCAGCCCCTATTGCGAGAATAGTCTATTATGCTACTGCAAAGAGATGACA
GCTGCATTTGTGTCGGCGCTGGACAGCGCCGTGCAGGACGAGATGGATGTAGCGGCGCAG
GTTTCCGACGAGGAGTGGGAAGCCATTAGGCCCAACAAATTGCAGAGAAGGGACTTTCTT
CTCCGCCATATGCAGCAACATATTGCGGATCACAACCGCTGCGGGAACTGCAGCACGTGG
CGACTGCGCGCGGGCGACACGTGGGGCGCCACCTACCGGCTCTACGAGGACCCCACCGCT
AACGAGACCGATGACAGTATTACTGATAACACTATCAAGGGAGCCTCTTTACAGCAGAAC
CTGCTGCAGGTGGGCTACTGGCGGCCGGTGGACGGCCTGAGATTTAGCGACGTCCTGTTT
CCGCACATCGAGCACGGCTTCCGGGGAAAGGTTATGCCGATCATCACGTTTCACAACCCG
CCGTGGAGCATTCTGCAGCTTAACGAAACTGGTTCTGTCACGAACTACACCGGCTTGACC
TTCGACATACTCCATCAGTTGGCTAAGAATAAAAATTTTACGTTGGGTCTCCTGTTCCCT
AGTGACATTAAGCAGGATACCAATGATACGATCAAAGAAGGCATACTCACGGCGGATGCA
ATAGCGTCGGCGCTGGCTGTGTCGCGCGGGCAGGTCGCCATAGCGGCGGCCGCCTACACC
GTCCTCAGAGAATCTATTGCCGGCACGAACTACACTAGCGTCATAAGTACGCAGCCGCAC
GCCTTCATCTTGGCGAGACCTCGCGAGTTGAGTCGCGCTCTGCTCTTCATGTCGCCGTTC
ACGTCTGACACTTGGCTCTGCTTAGGCTTCGCAGTTGTGCTGATGGGACCTACGCTATAC
GTTATACATCGTCTCAGTCCATTTTACGCGGAGAACAACTTGAACAACAGGCGGGGAGGT
CTGTCGACTATTCACAACTGCCTCTGGTACATGTACGGAGCCTTGTTGCAGCAGGGCGGC
ATGCACCTCCCCCAAGCCGACAGCGGTCGGCTGGTGGTGGGCACGTGGTGGCTGGTGGTG
CTGGTGGTGGTGACAACATACTCGGGTAACCTGGTCGCCTTTCTCACATTCCCCAAAATC
GAGGTGCCCATTACCACCATCGCCGAGCTGCTGAGCCACAGCGATACCATTACCTGGAGT
CTGAGGAAGGACACGTATTTGGAGATGCAGTTGAAGGAATCTCTGGAACCAAAATACAAA
GCACTGTTAGCTGGCGCACGGCTCCTGCCCCGAGGGCACCTGCCGATGGAGGGCGCGGGC
GATGCGGAGACGGAACGCGTGCGTCGCGGCGGCCACGTGCTGCTCGACTGGAAGATGCGA
CTCCACTACCTGATGCGGCGCGATCATCTGGAGACTGACACCTGTGACTTCGTACTCAGC
TTGGATGAATTCATGGATGAGCAGATGGCGATGATAATGCCTGCAAACAGCCCGTATCTT
CCAATAATTAATGAAGAAATAAAGCGCATGCACCAGATGGGCCTGATCCACAAGTGGCTG
TCGCAGTACCTGCCGCGGCGCGACCGCTGCTGGAAGAGCTCAACCAAGCTGACCACGGAG
GCGAACAACCATACCGTCAACATAGACGACATGCAGGGTTCATTCTTCGTACTCATCTTG
GGTTTTCTGACATCAGCATTCGTTTTGGTGATAGAATGGTATTATATGCGGTGGAGGAGG
AGGCGCGAACAGACCATCATCAAACCGTTCGTTCAATGA

EsigGR4

CAAGGCGTCCAGGACACACTGCTATCCATCGACATGCTGACAGCCGATCGTCCCACGCAG
AAAGAGGTGGATCATTTCATCCAGGCAATCGAGATGAACCCCGCCATCGTGAGCCTGAAA
GGCTACGCCCACGTCAACAGGGAGCTCTTGACATCGGCCATGAGTACGATAGCCATCTAC
CTGATCGTGCTATTACAGTTCAAGATCTCCTTGCCGAAGGAAGCC

EsigGR5

CCGGCGGTGGAGCTGCTGCACTACCAGCAGGTGCTACTTCGACGTGCTGCGCGCGCTCTC
GACGGCGGCTGCGGCCTCGTCAACACCGCCGCGCTCATCTTTTGCATCGCCGAGATTACT
GTTTCGATCTACTACACTATTACCGATTTGGGTTTTCGTCACATAAATTCGATGATTGTA
AACGTACAATGGATTGCCATGCACGCGGCGACCCTTATTCTGCTGGTGGAGCCGTGCCAC
AAGTACGGTTTGGAGGATTTGGCAATAAGCCGAAAACTGGAAATGATAGTGCTGCGCTTC
GAGTTGGCGATGGCTGGCGACTCTTACTGGCCGCGCGAGCGTCGGGTCAACGCCTTCTGG

CGTCGCCGGCCCACTTTCCGCGCCGCCGCCGCCGCCCTCAGCGCTATGGGCATCTGCGTA
CTGCAAAGAAAATTACTGCTCTCGATAATATCAACGGTAACAACATATCTCATCATTTTG
TTACAAGTGAAAGAC

EsigGR6

AGGCAAAATCTTAAAAAAGGAGACTTAGTTCTCATAAACTCCGATGCACCACCTTTGCAG
TGGCCGTTAGCGCGCGTAGTACGACTACATCCCGGCCCTGATGGCACCGTGCGCGTCGTT
GACGTTCGGACCACGAACAGCGAATTCACTCGACCTGTTTCAAGGCTCAGTCCTCTGCCA
TTAAATGAGGACGAAGATATAGCTTAG

EsigGR7

ATGAGGAGACTATTGCATAATAATCCGGTGCAAGAAAAGAAAGTGAACGACGGAAGTCCT
AAACCAACACCGAGTAATTCGTTCAACATAAGCGGAGAGCTGATGGTATTTGTGAAGGTA
CTGCGGGCGGTGGGCATGGCCCCGCTGCGTTGTGAGCAGGAGTCCACTGGGCGCTGGCGC
ATCACCACGTCCCTGGCGCTGCGGTTCTACAGTTGGATATTTATATTCATTGTTGTGTTG
GTGTCGACATACGCATTCTACGTACAGACGACACTGCCGGAGAAGGGTAGCATGGCCGCG
CGCCTCGTCAACCTGATGCAGTTCATCGCCATGAACTCCTGCCTCCTGGCCACCGTCTTC
ACCATGCCCCTCAGGACTAGGACACTCGTGGAGTATGTCGACACTATGTCCCAATTGCAT
GACCGCTTCAGCAAGGACGAGTTCAAGGTATCGAACTGCAGGATGCTGGGACTGCTGCTC
GTTTCCCTCTTGGCCTTCGTCGTGGTATTGGTGCAGGAAACCGCGGATAACGTACTCGAC
CAACCCGTTGTAGATGCAAAGACTATACTCTGCTACGTGCCGTCGCCAATACTGTACATG
TTGTGTCTAATGATGGAGGGGGAATTTACATTCCTGGTTTGGCTGCTGGAGAGTCGCGCT
GAAGCCGTCATCAGGCGAATCGACAACTTCCTCAAGCAAGGAGAGAGGCACAGGTGGCGT
GACACCAACCATGTGCTGCAACGCATGCAGCAGCTACATGAACTGCAGGTTGCGCTGCGA
AGGTGCGCTGGCGTCATCAACGCTGCCTTCGGCCTGCCCATGCTCATCGCTGTTGTTTTC
ATCATGATCGAGATCATCATCGCCATTTACTATATGATCGTTGATCTAGATCATTTTGGT
GTTCGAACTTTTCTGTTGAACCTGCAATGGATTTTGATTCTGGTGTGGTGGCTTATGGTG
GTAATAGAACCTTGTCACCAGTACGTTGAAAAGGATCAGGTAATATCTAATCGAATCAGC
AAAGTGATCCTGAAGCTAAGCGAGTCTAAAATCTTCAACTGCGACTGGCGCGACGAGAAT
CAGATCAAGATCTTTAGGGCCCAGCAGGTCCTCTTCAGCAGCACGGAGGTCAACATTACG
GCACTCGACGTCTGTGTCATGAGAAGATCTCTCTTGGGATCGATCGCTGGAGCTGTTACT
ACTTATCTTGTCATTCTGCTTCAGTTAAAAGATACAAAAATTTAA

EsigCSP1

ATGAGTCCATTGACAGTGGCGTGCCTACTAGCGGCAACATGTTTTGTGCACGCGGCCCCG
GAACACTATACGAGCAAGTACGATGGCATCAATTTGGATGAGATCCTCGCCAACCGGCGC
CTCTTCGTGCCCTACATCAAGTGCATCCTCGACCAGGGCAGGTGCACTGCCGAGGGCAAG
GAGCTTAAAGCACACATCAGCGATGCGTTGCAGACGGGCTGTAGCAGCTGCACGGCGACG
CAGCGCAAGGGCGCTCACAAGGTCATCGGACACCTCATCCACCACGAAGGCGAGTTCTGG
CAGCAGCTGGTTGACAAGTACGACAAGACGCGCCAGTACACGCGCAAGTACGAGCACGAG
CTCAAGAGCATTGCCGCGTAA

EsigCSP2

ATGTTACTGCGTAATAGAGTGGGTGTGGTCGCGCTGGTGGTGCTGGCGGCGGCGGCAGTG
GCCACGGCGGCGCAGACCCGCTACTCGGAGCGCTACGACCAGGTGAACGTGGGCGAGGTG
CTCGCCAACCGCCGCCTCTTCACGCCCTTCGTCAACTGCATCATGGACCGCGGCCGCTGC
ACCGCACAGGGCCGCGAGCTAAAGAACCACCTGGCGGACGCGCTGCAGAGCGGGTGCGCG
CGCTGCTCGGCCAAGCAGCAGGCGGGCGCCACGCTCGTCATCCAGCACCTGATCCACCGC
GAGCCGGCCGTGTGGCGTGAGCTGACGCGCAAGTACGACCCGTACGCCATCTACCGCAAC
AAGTACGAGGCCTTCGCCGCCTCTAAAGGCATCACGCTGCCC

EsigCSP7
ATGAAGTCGACTCTTTGCATTGCGATACTAGCATGTGGCGTTCTAGTGACGGCTCAGTTC
TACAGCTCAAAATTTGAGACCATCAATGTGCAGGAGATTATAGGCAACAAGCGCATCCTT
CAAAACTACATCAACTGCTTCCTCGACGCCGGCAACTGCGCACCCATTGCTAAGGAATTG
AAAGATCTGTTGCCGGAAGTACTGGCGACGACATGCGGCAAGTGTTCCCCGAAGCAGAAG
GAGATCCTCCGCGGACTGTGCTGCGCCCTGCAGAAGCAGGTGCCCGCCGACTGGGCCAAG
ATGCAGAAGAAGTATGACCCCGGTAGCAAGTATCAAGTCTCCTTCAATAAGTTCCTCAAC
TGCTAG

EsigCSP9\11
ATGGGCGTGTACGCGGCACTGGTGGCGGCGGCTCTGTTCGCGTGCGCAGCGCGAGCACAG
CGCGACTCGCGCTACGACTCCCGCTACGATTACCTGGATGTCGACGCCATCCTCGACAGT
CGCCGCCTCGTGCGCAACTACGTCGAGTGCCTCCTCAACAGGAAGCCCTGTTCACCGGAG
GGCAAGGCGCTCAAAAGAATATTACCAGAAGCCCTTCGCACCAAGTGTGCAAGATGCACT
GAACGCCAGAGAGAGAGCGCAGTGAAAATTATCAAAAGACTGAAAAGTGATTACCCGGAC
GAATGGTCAAGGTTGGCGGGCCGTTGGGATCCCAATGGAGACTTCACAGCATATTTTGAA
CATATAGTTGCCAAGGAGGACAAGAACCAAATATCGGGCAGCAAATTAGTACCTCAGGCC
ACTGGAATTGCTATAACAAATATCACTGCTCCAACAAATTTAGTGCCTTTGCGACCTATT
GTACTAACAAGGTAA

EsigCSP10
ATGTTCGGCCAGGTAATAGTGGTGTTATGGATGCTGGCGTACGCGTACGCGGGGAGCGGC
GAGGGATTTTCCACGAAATACGATAACGTCGACCTTGACCAGATCCTCACCAACGAGCGC
CTGCTCACCAGCTACGTCGCCTGTCTCCGGGACCAGGGTCCGTGCTCGCCCGACGGCAAC
GAGTTGAAGAAGAACTTGCCGGAAGCGGTGAGGGATGACTGCAGACGATGCAGCGAAACA
CAGCGGCGCGGCTCGGCGCGGGTCATGCGCTACCTCATCGACCACCGGCCTGAGGACTGG
ACACAGCTTGAGAACTTATACGATCCAGAAGGTCTGTATAAGAAACGTTACTTAAAGAGC
GCAGAGCTCGCCACAAACGAAGAGGAGAATTCCAGCCCGACTACTGAGAAGGCCTGA

EsigCSP12
ATGCAGCCCACAATTCTGTTTATCTGCTGCGTCGTCATCGCTGCATCAGCTTCAGCAGCT
GTAACACCACGTCCCGCCGTATCCGATACAGCACTTGACAACGCGCTGCAGGACAAGAGA
TACCTCATGAGACAGCTTAAATGCGCTTTGGGTGAGGCACCTTGTGATCCCGTTGGGAGA
AGATTGAAAAGCTTAGCTCCATTGGTACTTAGAGGTTCGTGTCCTCAGTGCACGCCGCAG
GAGATGAAGCAGATCCAGAGAACACTGGGACACGTACAGAGACATTATCCCAAAGAATGG
GCTAAGATGCTCAAACAGTATGCCGGAAAGTAA

EsigCSP13

AACGTGACCAACGTGCGCAGCCTCGTCTACTGCTTCCTCGAGGAGGGCCCTTGCGATCCC
TTCGCCAAACACATGCTAGACATCTTGTGGGAGGCGGTAGAGACAGAGTGCACGAAATGC
AATCCGAAGCAGGTGGAGCAGTACGAGAAGGCGATGATTGCTATATACAAGCTGTATCCC
AAGGAACACAAGAGAATTGAAGACAAGTATGACCCGGAGAACAAGTGCACTGCCATTAAA
GCTTTGTTAGAGAAAGCCATTGATTTATGCGTAAATTAA

EsigCSP14

ATGAAGGTAGTTATCGTCCTGATGATGTGCGCGGTAGCGGCTCTATCAGCCCCGGCCGAC
AGTCCTGAGAAGTACACAGACCGCTACGACAACATTAACCTAGACGAGATCCTCAGTACT
AAACGTCTGCTAATCGCCTACGTCAAGTGCGCCGTCGACAAGGGCCGCTGCACGCCCGAG
GGGAAGACCCTCCGAGATCACCTTGAAGACGCACTGAAGACTGGCTGCGCCAAGTGTACG
AAGGCGCAGGACGAGGGCGCCGACCGCGTCATAATTCACCTCATCGAGCACGAGCCCGAG
CTCTGGAAGGAGCTGGTCGAGTTCTACGACAAGGACGGTACTTACAGGAAGACTTACGAG
GACCGCGCACGCGCCAAGGGCATCACCATCCCTGCATACTAA

EsigCSP15

ATGTTGGTCGTCGTAGTGGGAGCGTTCGCCATTGACACGGCCTTCGACGACTACGACGTA
GACAGTTACGTGGCAGACGTGACCAGCGTGCGCAGCTTCGCCTACTGCTTCCTCGAGGAG
GGCCCTTGCGATCCCTTCGCCAAACACTTGCGAGACAACTTCTGGGAGGCGGTAGAGACA
GAGTGCAAGGTATGCAATCCGAAGCAGGAGGAGCAGTACCTGAAGGTGATAGCGGTTATA
TACAAGATGTATCCCGAGGAACACAAGAGAATTGCAGACAAGTACCGGTATGACCCGAAG
GAAAAGTACCGCATTGTCAATTAG

EsigCSP16

ATGGCCTTAGAGACGTTTTCACTCGAAAATCCACTTTTCCGTACCTATCTTGTTTACTCA
TCAATTCTCGTTTTAAAAATGTTGATCATGTCTGTTTTGACCGCACGGCAGCGTTTTAGG
AAAGGGGTATTTGCCAATGCCGAAGACGCTAAAACTCCAAAAGCAAAGGTCAAGTACGAT
GATGTTGACGTGGAACGTGTTCGAAGAGCTCATCAGAATGACTTGGAAAATATTCCAATC
TTCCTCGTGATAACTTCCATGTATATACTGATTGATCCACCAGTATTCCTAGCGACCAAC
CTGATCAGGTTGTTCACAGCAGGACGAGTGCTTCACACTTTGGTATATGCTGTCTGTCCC
GTTCCACAGCCTGCACGTGCTTTGGCTTGGGCTGCTGGTTATTTACCACTATTTTTATG
GCCACTTCCGTATTTATAAAATTTATATCTGATTTGTAA

EsigSNMP2

ATGAAGCTCCAAAGGCACATGAAGGTGGGACTTGGTTCGGGCGGTGCGGCCGCGTTCGGC
GCCCTCTTCGGCTTCGTCGCCTTCCCCGCCCTCATAAACAGCGGTCTTAAGAAGGACATG
TCGCTCTCCGAGAAGAACGATATCCGCGGCATGTGGAGCAAGGTGCCGTTCGCGATCGAT
TTCAAGGTCTACCTCTTCAATTGCACCAACGCTGAGGAGGTACAGAAGGGCGCCACGCCG
GTGCTGCAGGAGGTTGGGCCTTACTACTTCGAAGAATGGAAAGAGAAGGTGAACCTGGAA
GACGTGGAGGACGAGGACGCCGTCATCTACAACCGCAGGGACACCTTCTACTTCCGGAAG
GAACTATCAGGGCAAGGACTGACTGGAAACGAAATGATAGTCATGCCGCACGTGATGATG
CTGTCATTGGCCATAGTAGTATCAAACGAGAAGCCGGCCATGCTGAATATGGTCGGCAAA

GCCCTCAACGGAATATTCGACGAGCCAAAGGACGTATTCCTGCGCGCGCGCGCCATGGAC
ATCATGTTCGACGGCGTGGTCATCAACTGCGCGAGGACAGAGTTCGCGCCCAAGGCCATC
TGCACAGCCATCAAGAAGGAGGCCAGCAATCTGGAGTTCTTGCCTGGAGACCAGTTCAAG
TTCTCGCTGTTTGGGATGCGCAACGGTACCACGGACAATAAGGCCATAAAAGTGCGTCGC
GGGATCAAGGACATTATGCAGGTGGGCCAGGTGTTCGGCGTGGACGGCGAGGACGAGCAG
AGCGTCTGGGCGGGGGAGCCCTGTAACCAATTTGCCGGTACCGATGGTACCATTTTCCCG
CCCTTCCTCACTTACAAGGACAAGCTGGAATCGTTCTCGCCCGATATTTGCAGAACTGTT
CGCCCCGAGTTCCAGAAGTTTACCTTGTACAAAGGCATTCCGACGAACCGGTACGTCGCC
ACCCTGGGTGATCTCGGCAACGATCCAGGCCTCAGCTGCTTCTGCGACGCGCCCGACAAG
TGCCCTAAGAAGGGTCTCATGGATCTCTTTAAATGCATAAAGGCACCGTTAGTTGCATCT
CTACCGCATTTCCTGGAGACGGACCCGTCGGTGTTAGCTGGAGTCAAGGGTCTGCATCCG
AACGCTGAGGAGCATAGCATCTATATCGACTTCGAACCGATAACGGGTACGCCGATGGTT
GCACGACAGCGCCTCATGTTCAGTATACAGCTGAAGACTATCGGCAAGCTGGAGCTTTTC
AAGGAACTTCCCGGCTCGCTGGTGCCACTGTTCTGGGTTGAAGAGGGTCTGGCCTTGAGT
GAGGGCTTCGTCGGTGTATTAAAATCGCATCTCTTCACACCGAAGCGCGTGGTCGGTGTC
ATGAAGTGGCTTCTGGTCAGCTTTGGCTCTTTGGGCCTCATCGGTTCTGCGCTGGTGCAT
TATAAGGACAAGATAATGAAGTTCGCCATCGCGCCTGACGCTCCCGTGTCCTTGCAGAAG
AAAGAAGGAAAGGAATTAAGTACTATAAGCAATTAA

EsigSNMP3

ATGTGGATCTTGCTAGGATCTGTGGCGTTCCTCGTTTTCGCCATAATAATGGCAGCCTCA
GGATTTCCGAATTTGGTTAAGCAACAAGTTAGTAAGGAGTTGGCTATAAGAAACGACTCG
TCAAAGTTTGAGTTCTGGCGTAAACTGCCCTTGCCGCTCAGCTTCAAAGTCTATTTGTGG
AACGTGACCAATCCGGAGGAGATCACGGCCGGCTCTAAGCCAAAACTAAAGCAGGTCGGC
CCCTATGTTTACGACGAATACAGAGAAAAGACTGTTTTGGGTTATAGAGAAAATAACACA
GTGGAGTATACCGAGAAGAAAACATACATTTTTAATAAAGAGAAGTCTGGAACGTGTACT
GAAGATGATCAAGTAACTGTCATTAATCTCATATTTATGACAGTGATGCTGAAACTAAAT
GAACTGATGCCATCTATGATGACCATGCTCTACAAGGCGATGCCGCTTCTTTTTACCGAC
CTGAAGGATCCCTTCTTGCGTGCTCGTGTCGGCGATATACTTTTCAATGGAATATTCCTC
GATTGTGGAGGCAACGAGACGACTCTCAAACTGGTATGCGGTCAAATTCAGGACGAGGCA
GTGCCAACTTTGAGGAAGGCCGATGATGGCAGAAATGGATTTTTATTTTCCATGTTTCAT
CATCTTAACTCGACGCCTGCCGGTCCTTTCCGCGTGGATCAAGGGGTCAAGAATATTAAG
GACGTTGGTCGAATAGTGACTTACAAGGGCGAGCCGGACATCGACATATGGAGCGAGCCG
ACCTGCTCCATGATCAACGGCACAGACGGCACCATCTTCGCGCCGATCGATCGCTCCAAC
ATTACTGATAAGCTGTACTTGTTTCAAGGAGACATATGCAGATCGATCTACATATCGCTC
TTTGGCGAAACAAAAACTTTCAACATGCCTACTTTTCGATATGAAAACACCAGAGAATTG
TTTGCGTCAAAGAGTGCCGTACCTTCCAACAAGTGCTTCTGTAAAAAAAATTGGATCAGT
AACCATGATGGATGTCTAATGAATGGCGTCCTCGATTTGGAGATGTGTCAGAAGGCTCCA
GTAATGGCCAGCTTCCCCCACTTCTATCTAGCTAGCGAGGAACTCCTTGAGTACTTCGAG
GAAGGAGTGGAACCAGATAAAGAAAAGCACGGTAGCTTTGTGGAATTAGAGCCGATTACC
GGTACGGTATTGCGTGGAGCACAGAGAGTTCAATTCAACATAGAACTGCGCAAAATGGAA
AATGTTCCTATCCTGGACTCCGTTCCAACTGGGTTATTTCCGTTGCTATGGATTGAGGAG
GGTGCCGAAGTAGATGACAAAATTATAGCCATGCTAAGCGACGTACACCGCGCGCTGCGC
TACGTGGAAGTTGCTCGTTGGGTCCTGCTAGCTGCAGGCTTGGTCGCTGCTCTCGGCGCA

GGCGTCGTCTACATTCGTCGCCACTACGCCCTCTGTGGAGCTACAAGCAGCGTCACCTTC
GTCGTGGATCAGCACCAGCTTAATGGCAATAAGAATCTAGCTTTCGTCGAAGACAGTAGG
AGCATGTAG
