# Supplementary material for: A Study of Adult Olfactory Proteins of Primitive Ghost Moth, Endoclita signifer (Lepidoptera, Hepialidae)
Source: Life (Basel). 2023 Nov 27;13(12):2264. doi: 10.3390/life13122264 (PMC10744962; doi:10.3390/life13122264)
Supplement: Supplementary file 1 [file life-13-02264-s001.zip › Supplementary file S2 edit1.pdf]

A Study of Adult Olfactory Proteins of Primitive Ghost Moth, *Endoclita signifer* (Lepidoptera, Hepialidae)

Guipeng Xiao [1], Jintao Lu [2], Zhende Yang [2], Hengfei Fu [2,*] and Ping Hu [2,*]

Additional file S2 The protein names and gene accession numbers were used in phylogenetic trees

OBPs

OBP		OBP		OBP	
**Accession NO.**	**Name**	**Accession NO.**	**Name**	**Accession NO.**	**Name**
AAF06123.1	SexiPBP	AAF06124.1	AvelPBP3	ABY71034.1	PxylGOBP1
AAF06144.1	CmurPBP4	AAC36315.1	HzeaPBP	ABY71035.2	PxylGOBP2
AAF06143.1	YcagPBP	BAF64703.1	AselPBP2	ACI28451.1	PxylPBP1
AAF06142.1	SexiPBP	AAX85460.1	AsegPBP2	AEB54587.1	HarmOBP6
AAF06141.1	PgosPBP	AAX85459.1	AipsPBP2	AEB54584.1	HarmOBP4
AAF06140.1	CrosPBP1	AAX85458.1	AipsPBP1	AEB54582.1	HarmOBP3
AAF06139.1	CrosPBP2	AFD34183.1	AconPBP2	AEB54581.1	HarmOBP5
AAF06138.1	CpinPBP1	AFD34183.1	AconPBP3	AEB54580.1	HarmOBP1
AAF06137.1	CrosPBP	AFD34176.1	AconPBP1	CAC08211.1	HarmGOBP2
AAF06133.1	CparPBP2	ABY71034.1	PxylGOBP1		
AAF06132.1	CmurPBP1	ABY71035.2	PxylGOBP2		
AAF06131.1	CmurPBP2	ACI28451.1	PxylPBP1		
AAF06130.1	CmurPBP3	AEB54587.1	HarmOBP6		
AAF06129.1	CfumPBP1	AEB54584.1	HarmOBP4		
AAF06128.1	CfumPBP2	AEB54582.1	HarmOBP3		
AAF06127.1	CfumPBP3	AEB54581.1	HarmOBP5		
AAF06126.1	AvelPBP1	AEB54580.1	HarmOBP1		
AAF06125.1	AvelPBP2	CAC08211.1	HarmGOBP2		

Orco

Orco		Orco		Orco	
**Accession NO.**	**Name**	**Accession NO.**	**Name**	**Accession NO.**	**Name**
NP_001296031.1	PxyOrco	XP_039752153.1	PageOrco	XP_014363049.2	PmacOrco
NP_001299600.1	AtraOrco	QRF70964.1	ScinOrco	XP_045516753.1	PbraOrco
XP_021195606.1	HarmOrco	XP_034828070.1	MhypOrco	XP_045503131.1	CcroOrco
XP_053617456.1	PintOrco	XP_038212518.1	ZcesOrco	XP_045486972.1	PrapOrco
XP_023942390.2	BanyOrco	XP_030036707.1	MsexOrco	XP_045453871.1	McinOrco
XP_052751921.1	GmelOrco	GBP69367.1	EjapOrco	XP_032527723.1	DpleOrco
XP_050667777.1	LsinOrco	XP_028178675.1	OfurOrco	ARO76408.1	CpunOrco
XP_050552928.1	SfruOrco	XP_028043387.1	BmanOrco	QKZ93458.1	AlepOrco
XP_050353359.1	NioOrco	XP_026726946.1	TniOrco	ON420156	MlorOR33
XP_049879122.1	PgosOrco	XP_026490757.1	VtamOrco	QNS36226.1	MsepOR33
AKW50880.1	EobiOrco	XP_026333801.1	HkahOrco		
WCC57650.1	PxutOrco	XP_022831582.1	SlitOrco		
WCC57583.1	PpolOrco	AJF20962.1	ObruOrco		
WCC57335.1	PdarOrco	XP_047538427.1	VataOrco		
WAW99989.1	SfurOrco	XP_047511461.1	PnapOrco		
XP_037974487.1	PxylOrco	XP_047033069.1	HzeaOrco		
QZH55165.1	AyunOrco	XP_046963887.1	VcarOrco		
XP_041972175.1	AageOrco	XP_045770122.1	MjurOrco		
